# Smart Continence Care for People With Profound Intellectual and Multiple Disabilities Within Dutch Residential Care Facilities: Economic Evaluation Alongside a Cluster Randomized Trial

**DOI:** 10.2196/72017

**Published:** 2025-10-10

**Authors:** Vivette JC van Cooten, Ghislaine APG van Mastrigt, Andrea Gabrio, Silvia MAA Evers, Marieke FM Gielissen, Brigitte Boon

**Affiliations:** 1 Academy Het Dorp Research & Advisory on Technology in Long-term Care Arnhem The Netherlands; 2 Tranzo Tilburg School of Social and Behavioral Sciences Tilburg University Tilburg The Netherlands; 3 Department of Health Services Research, Care and Public Health Research Institute (Research Institute CAPHRI) Faculty of Health Medicine and Life Science Maastricht University Maastricht The Netherlands; 4 Department of Methodology and Statistics, Care and Public Health Research Institute (Research Institute CAPHRI) Faculty of Health Medicine and Life Science Maastricht University Maastricht The Netherlands; 5 Centre for Economic Evaluations Utrecht Netherlands Institute of Mental Health and Addiction Trimbos Institute Utrecht The Netherlands

**Keywords:** economic evaluation, cost-effectiveness, cost-utility, profound intellectual and multiple disabilities, continence care, technology, eHealth

## Abstract

**Background:**

Persons with profound intellectual and multiple disabilities in residential care facilities may benefit from smart continence care (SCC), which is incontinence material (IM) with integrated sensors that notify caregivers when the IM is saturated and requires changing. SCC aims to reduce weekly leakages and improve the quality of life.

**Objective:**

Given the growing demand for health care services and the decreasing workforce, it is essential to assess the cost-effectiveness and cost-utility of such technologies.

**Methods:**

This economic evaluation was conducted alongside a cluster randomized trial across 6 care organizations in the Netherlands. The incremental cost-effectiveness ratio (ICER) was expressed as the additional societal costs of SCC in relation to a reduction in weekly leakages. The incremental cost-utility ratio used Quality Adjusted Life Years measured via the EQ-5D-5L proxy 1 version. Robustness was assessed using bootstrapping, sensitivity, and subgroup analyses for variations in pricing and alternative outcomes (weekly IM changes [IMCs] and time savings). The study period was 12 weeks.

**Results:**

The analyses included 74 participants in the regular continence care (RCC) group and 82 participants in the SCC group. Analyses were corrected for baseline differences in the time spent on continence care and utility. SCC is found to be less effective (–1.058, 95% CI –1.878 to –0.262) and more costly (US $371, 95% CI US$ –0.09 to $771) than RCC for the number of leakages as the outcome, placing the ICER in the northwest (NW; inferior) quadrant of the cost-effectiveness plane. Cost-utility analyses showed a high uncertainty, with results in both the NW and northeast quadrants. Scenario analysis suggested that the negative effect on leakages was due to implementation challenges. Sensitivity analyses showed that the supplier’s new pricing model had a slight positive effect, reducing the estimated total societal costs, although uncertainty remains. SCC was estimated to effectively reduce weekly IMCs (ICER in the northeast quadrant) but did not save time (ICER in the NW quadrant).

**Conclusions:**

The results of this economic evaluation are not conclusive because of the mixed outcomes and a limited timeframe. SCC is ineffective in reducing the number of weekly leakages, but it does reduce the number of weekly IMCs. However, SCC was not effective in reducing time spent on continence care. Findings suggest that SCC is expected to be more expensive than RCC, although the supplier’s new pricing model may decrease costs. The use of technologies such as SCC should not solely be based on cost-effectiveness and cost-utility outcomes. This technology offers value by generating data that can support personalized care. However, the realization of this added value is not guaranteed and may differ between individuals. Implementation challenges and individual variability underline the need for tailored approaches.

**Trial Registration:**

ClinicalTrials.gov NCT05481840; https://clinicaltrials.gov/ct2/show/NCT05481840

## Introduction

Worldwide, the estimates for the prevalence of persons with intellectual disabilities, an IQ below 70, vary between 1% and 3% [1-4], which is 80-234 million people. In the Netherlands, 2.3% of the population (around 400,000 out of 17.4 million individuals in 2024) has an IQ below 70 [5]. The severity of intellectual disability, as well as accompanying physical and medical challenges, can vary greatly, thereby influencing the type of support these individuals need. A specific group of persons with intellectual disabilities is individuals with profound intellectual and multiple disabilities who are fully dependent on others. In the Netherlands, there are approximately 10,000 individuals with profound intellectual and multiple disabilities; 90% (9000/10,000) of those individuals spend most of their lives within residential care [6,7], living together with other individuals with intellectual disabilities and receiving 24/7 care and support. Their disability is characterized by a profound cognitive disability in combination with physical disabilities and medical problems [8]. As IQ tests are not the most appropriate tool to assess their cognitive functioning, various definitions related to IQ scores exist (either below 35 or below 20). A commonly used description is a developmental age of less than 48 months [7]. Of all the expenditures in 2023 financed for the Dutch long-term care budgets (wet langdurige zorg), 36.6% (€11,481 million/€31,366 million [A currency exchange rate of €1=US $1.05 as of December 6, 2024, is applicable hereinafter]) was spent in disability care [9]. No figures are known for the specific subgroup of individuals with profound intellectual and multiple disabilities.

A significant portion of the support that persons with profound intellectual and multiple disabilities receive is spent on their personal hygiene, including incontinence care. Currently, it is common that the incontinence material (IM) of individuals with profound intellectual and multiple disabilities in residential care is changed according to fixed schedules rather than on demand when IM change (IMC) is actually needed. This may lead to unnecessary changes, if IM is changed too early, as the material still has spare capacity to contain urine, or to potential leakages of the urine outside the IM if the IM is changed too late, as the material is oversaturated, requiring changing clothes and possibly washing or showering the person. Both situations result in unnecessary resource use and costs, as well as possible personal inconvenience and medical complications, such as irritated skin when IM is often changed too late [10]. Providing more personalized care and ensuring timely changes may optimize continence care.

In current times, more and more technological applications are being developed and used within care organizations to optimize the care process. These innovations aim to support individuals with intellectual disabilities and their professional caregivers (hereafter: caregivers) [11-14], providing greater access to high-quality personalized care [15]. Besides the possible benefits care technologies can bring to a person with intellectual disability, caregivers may also benefit from these technologies, as this supports them in their work and saves time. With the decreasing number of caregivers and increasing demand for health care services that are currently occurring [16], time-saving technologies may help increase the sustainability of our health care system. Since health care budgets need to be spent wisely, it is important to study the (cost)effectiveness of technological solutions from a societal perspective [17,18].

Smart continence care (SCC) is such a technological solution that may improve the care for persons with profound intellectual and multiple disabilities, and on the other hand, lead to a more efficient use of caregivers’ time. IM containing sensors can notify caregivers when a change is needed [19-22]. A detachable clip transmits information about the saturation level of the IM to caregivers’ phones, indicating the need for change [23]. Although previous studies provide information about the potential benefits and costs of SCC [24,25], a large-scale study focusing on the added value of SCC for persons with profound intellectual and multiple disabilities is lacking. Therefore, a cluster-randomized trial (CRT) was conducted to evaluate the clinical effectiveness of SCC compared to regular continence care (RCC) [26]; results are reported elsewhere [27]. In addition, a trial-based economic evaluation was conducted alongside this CRT, which is presented here. The aim is to evaluate the cost-effectiveness and cost-utility of SCC versus RCC for persons with profound intellectual and multiple disabilities in the Netherlands from a societal perspective.

## Methods

### Study Design

The current economic evaluation was conducted alongside a CRT across 6 Dutch long-term residential care organizations for persons with intellectual disabilities spread across the Netherlands (hereafter: care organizations), which studied the effectiveness of SCC compared to RCC. Power calculations for the effectiveness study determined a sample size of 6 clusters with 24 participants each; we aimed for a total of 160 participants [26]. Randomization at the cluster level (1:1 ratio) assigned these care organizations to the waiting-list group (hereafter: RCC group), continuing RCC, or the intervention group (hereafter: SCC group), implementing SCC. Details on the recruitment and enrollment of care organizations, and the results are reported elsewhere [26,27]. The study was registered at ClinicalTrials.gov (NCT05481840).

During the study, a session was organized with the parents of some of the participants, in collaboration with a patient organization, to discuss the impact of (smart) continence care. This resulted in a better understanding of the complexity of the care process and that its potential benefits may differ from person to person.

During the study, data were gathered for this economic evaluation according to the study protocol [26]. The evaluation was conducted according to the Dutch Guideline for Economic Evaluations in Healthcare (EEH) [17] and reported according to the CHEERS (Consolidated Health Economic Evaluation Reporting Standards) reporting guidelines [28,29] (Multimedia Appendix 1) and CONSORT-EHEALTH (Consolidated Standards of Reporting Trials of Electronic and Mobile Health Applications and Online Telehealth; Multimedia Appendix 2). Data collection was conducted from September 2021 to April 2023 and occurred at T0 (baseline), T1 (6 weeks), and T2 (12 weeks) for both the SCC and RCC groups. In the original design, a T3 (follow-up) was added after 9 months. Due to time constraints, this T3 measurement could only be conducted in two of the three SCC (intervention) organizations. As time progressed, we found that care organizations’ boards required more time to decide on the continued use and upscaling of SCC. Postponing this decision by the boards resulted in the locations participating in our trial not using SCC at the time of T3. We thus decided not to include T3 measures in our economic evaluation.

### Participants’ Recruitment

Participants were enrolled by the care staff of the selected locations based on the inclusion and exclusion criteria [26]. The inclusion criteria were: a diagnosis of profound intellectual and multiple disabilities, older than 18 years, use of incontinence products, inability to communicate the need to change their IMs, and a signed informed consent by their legal representative. Exclusion criteria were the use of a permanent catheter or behavior that could be a risk factor for the participant when using SCC, such as a pica disorder that could cause the participant to swallow the clip. Inclusion was carefully considered for participants who defecated ≥3 times per 24 hours, as feces interfere with the technology detecting urine, and the presence of behaviors that could hinder the successful implementation of SCC, such as removal of the IMs or clips. The recruitment period of the study was from August 2021 to March 2023.

A total of 29 locations (residences) participated across the 6 care organizations throughout the Netherlands. These care organizations varied in size and organizational structure. The locations selected had multiple residents with severe intellectual disabilities in combination with physical disabilities or other medical problems. These residents received care and support throughout the day. The number of locations per organization varied from 2 to 7. The number of participants ranged from 2 to 12 per location, with an average of 5.4 participants per location. A participant received continence care from three different care teams during a week: (1) a care team from the residence, (2) a night care team, and (3) a day activity center’s team.

### Procedure

#### RCC

The RCC group received their usual continence care during the T0-T2 measurements. RCC involved changing the IMs of the participants by their caregivers according to their normal, mostly “fixed” schedules [7,11] or when they identified reasons for a change, such as detecting feces. Organizations assigned to the RCC group continued to use their regular IM brand. After the T2 measurement, the organizations switched to SCC for 12 weeks, as part of being on the “waiting-list design”; no data were collected in this period.

#### SCC

The SCC group received implementation guidance before and during the first 12 weeks of using SCC technology (T0-T2). At T0, the caregiver provided continence care as usual with their regular IM. Then, caregivers received training and started using the SCC solution. At T2, they used SCC for 6 weeks; at T2, this was 12 weeks. Caregivers used the SCC system of Abena Nova (Abena A/S, Aabenraa, Denmark) [26]. The product consists of an IM with integrated sensors, a detachable and reusable clip, receivers, a mobile app, and a web-based platform showing voiding patterns, IMCs, and notification data. An internet connection and mobile phones with the installed app were needed for caregivers to receive notifications. Depending on the degree to which the IM was saturated with urine, the notifications were green (no need for change), orange (change desired), or red (risk of leakage) [23]. Caregivers are requested to respond to these notifications by changing the IM on demand and stopping IMC according to fixed schedules. Thus, during the use of SCC, caregivers adapted their working routines in addition to using different IMs, sensors, and notification technology. After the T2 measurement, the organizations in the SCC group decided individually whether to continue or discontinue with SCC.

#### Implementation

Training and implementation activities were provided to prepare the care organization [26,30]. Each care organization established a project team with a dedicated project leader. Each care team appointed an ambassador as a liaison between their care team, other care teams, the project leader, and the supplier. Ambassadors received an explanation of SCC to help them understand the technology, and together with their teams and a specialist from the supplier, they selected the participants for SCC. The ambassadors received in-depth training from the supplier for approximately 2 hours. Next, the care teams received 1-2 hours of training from the supplier and ambassador. E-learning (30 min) was available as preparation preceding this training. Before and during the implementation process, the project leader received implementation guidance from the research team through weekly consultation sessions [26]. While conducting SCC, the care teams met regularly for evaluation. The information generated by the sensor was displayed in the web-based portal, showing the voiding pattern and indication of urine volume. The care teams used this information to fine-tune the SCC to an individual’s needs.

### Clinical Outcomes and Quality of Life

The clinical outcomes related to continence care were collected using continence diaries completed by caregivers for each participant at T0, T1, and T2, each over a 1-week period [26]. From these diaries, the number of weekly leakages, the number of weekly IMCs, and the duration of continence care can be derived. The clinical outcome for the base case cost-effectiveness analysis was the change in weekly leakage in relation to the change in total societal costs.

The outcome of the cost-utility analyses (CUA) was Quality Adjusted Life Years (QALY), and measured according to the EQ-5D manuals [31]. The paper format of EQ-5D-5L proxy 1 version was used, as participants with profound intellectual and multiple disabilities cannot self-report. Their professional caregivers acted as proxies. This instrument is considered valid and reliable for the general population and represents the standard health outcome reference in CUA [32,33], and is recommended by the Dutch guideline for EEH [17]. There is currently no scientific evidence on the validity of this approach for persons with profound intellectual and multiple disabilities. The EQ-5D questionnaire provides a health status based on five dimensions: mobility, self-care, usual activities, pain or discomfort, and anxiety or depression. Each dimension is scored on a 5-point scale, giving 3125 possible health states [34]. These health states are valued using the Dutch utility score [32], varying from –0.446 to 1, as recommended in the Dutch guideline for EEH [17], with negative values representing health states valued “worse than dead.” These are then aggregated to obtain individual QALY measures computed over the relevant study period (T0-T2) through an area under the curve approach [18].

### Costs: Identification, Measurement, and Valuation

#### Identification

To identify relevant cost components, the DIRUM (Database for Instruments of Resource Use Measurements; June 2021) was consulted. This database did not contain suitable instruments for collecting resource use data specific to continence care. Therefore, the relevant cost components were identified through a literature search, field observations, and a brainstorming session with employees within a care organization (such as a coordinator of night care, physiotherapist, and project leader) [26]. Furthermore, the Dutch guideline for EEH [17] provided input for relevant cost categories. This resulted in five main categories: (A) “Societal costs perspective,” consisting of the two main categories (B) “Total health care costs” and (C) “Cost for participant and family.” “Total health care costs” (B) consists of (D) “intervention costs” and (E) “other health care costs.”

A=B+C; B=D+E.

From a care organization’s perspective, the intervention costs (D) are most relevant when deciding to implement SCC, as they directly influence their income statement. The relevant cost components identified during the brainstorming session were: costs for staff providing continence care, IM and disposables, wound and skin care, laundry, waste disposal, and the cost for the SCC system and hardware. Waste disposal costs were excluded from the analyses, as accounting for these costs from a sustainability and carbon footprint perspective would also require considering the environmental impacts of the necessary IT services, software, and additional hardware. Conducting a thorough assessment of these factors demands specialized expertise, which is beyond the scope of this study.

The cost aspects relevant to other health care costs (E) were derived from the Dutch guideline for EEH and based on the iMTA Medical Cost Consumption Questionnaire (iMCQ) [35]. This questionnaire collects information on resource use of other health care services, such as questioning the number of visits to a general practitioner, physiotherapist, or outpatient clinic. Due to time constraints, the use of medication, except for wound and skin care related to continence care, was excluded from this study. Although including medication costs is of interest for the health care costs for persons with profound intellectual and multiple disabilities, for example, when reporting on the burden of disease, it was not expected that the medication regimen would change due to a change in how continence care is provided. The cost items for the participants and their families (C) were limited to travelling costs, as individuals with profound intellectual and multiple disabilities are not employed or do not do voluntary work. Productivity loss for family members was deemed not relevant as their relatives are living in residential care.

#### Measuring Resource Use

The resource use related to RCC and SCC was gathered with the continence care diaries. Besides collecting data about the number of leakages and IMCs, the time per IMC was registered. This included the additional time needed for laundry or other personal hygiene activities if a leakage had occurred. Furthermore, the diaries collected information about the amount and type of laundry that resulted from a leakage, as well as the number of times wound and skin care were provided. The duration of continence care for the period of interest is calculated by taking the average time of one IMC multiplied by the weekly number of IMCs and then multiplying by the number of weeks of the preceding period.

The resource use relevant to “the other health care costs” was based on the relevant items of the iMCQ with a 12-week recall period [35]. Caregivers completed the iMCQ questionnaires as a proxy and could refer to the electronic patient file for verification. This follows the Dutch guideline for EEH, which recommends this as a primary data source. Secondary data sources, such as claims data, are not used. The selected items can be found in Table S1 in Multimedia Appendix 3. The answers to this questionnaire also provided input for calculating the travel costs (patient and family costs). Table 1 gives an overview of the measurements and the research instruments used in this health economic evaluation.

**Table 1:** Overview of the questionnaires.

Outcome (research instrument)	T0 (week 0)	T1 (week 6)	T2 (week 12)
Baseline characteristics (paper questionnaire)	✓		
Information on continence care and resource use (1-week paper continence diary)	✓	✓	✓
Health care resource use (iMCQ^a^)			✓
Quality-adjusted life years (EQ-5D-5L proxy 1 version)	✓		✓

^a^iMCQ: iMTA Medical Cost Consumption Questionnaire.

#### Valuation

Prices for the variables in the category “other health care costs” were derived from the Dutch guideline for EEH [17] in order to determine their costs and adjusted to 2022 Euros. Travel costs were calculated by multiplying the average distance by standard price weights for taxi use provided by the Dutch guideline [17]. For cost items lacking prices in this manual, valuation was based on literature research and organizations’ invoices; more details can be found in Multimedia Appendix 3. Discounting was not necessary since the measurement period of the study did not exceed 1 year (discount rate 0%).

### Health Economic Analyses

#### Base Case Analysis

The primary (base-case) analyses were performed according to the intention-to-treat principle, using linear mixed regression methods via R software (version 4.0+; R Foundation for Statistical Computing) and using the nlme, lme4, and BCEA packages for both outcome and cost measures, computed over T0-T2. The model accounted for the clustering of the data (at the location level) while also controlling for potential imbalances between arms in the following baseline variables: the average time spent on continence care at T0, the number of weekly IMCs at T0, and the health-related quality of life (Dutch utility score) at T0. The technical details on these models can be found in Multimedia Appendix 4.

In all analyses, missing effectiveness and cost values are addressed through multiple imputations using chained equations under the assumption of a missing at random [36]. An imputation model for each outcome of interest is specified, including all predictor variables used in the main analysis, as well as variables that are found to be potentially predictive of missingness according to preliminary analyses. A total of 10 imputations are generated for each missing value via a predictive mean matching approach [37] to ensure that key characteristics of the data (eg, skewness) are preserved when imputing, while also accounting for the clustered nature of the data at the location level. Each model is then fitted to each imputed dataset, and combined estimates of the parameters of interest across imputed datasets are derived using standard multiple imputation rules [36].

In the case of missing days within the continence care diaries, the number of registered weekly leakages and IMCs was extrapolated to the total number of weekly leakages and IMCs, following the same approach used in the effectiveness study [27]. That is, for example, if two leakages had occurred over 5 days, while 2 days were not registered, the number of leakages was set to 2/5×7=2.8 for that particular participant at that time point. If weekly leakages and IMCs cannot be extrapolated due to a missing measure, thus a completely missing week, then this is handled via multiple imputation methods as described above.

Following standard practice for trial-based economic evaluation, after imputations are generated, results from each analysis model are combined with nonparametric bootstrapping methods (with the number of replications set to R=2000), to generate estimates that are robust against typical features of cost-effectiveness data (eg, skewness) and to quantify the uncertainty associated with the results [17]. Results from all analyses are reported in terms of estimated means, SEs, and associated 95% bootstrapped CIs constructed based on the empirical percentile method. Cost-effectiveness planes (CE-plane) for all clinical outcomes and utility and cost-effectiveness acceptability curves (CEAC) for only utility are used to visually represent the uncertainty around bootstrapped replications of key estimated quantities for the economic evaluations (eg, incremental cost-effectiveness ratios [ICERs]).

The CE-plane shows differences in effect on the horizontal axis and the differences in costs on the vertical axis. Each bootstrapped result is then plotted on the CE-plane and reflects the difference in effect and the difference in costs. As imputation and bootstrapping result in multiple outcomes, all these outcomes are plotted on the CE plane, resulting in a “cloud” with results. Results in the northwest (NW) quadrant mean that the intervention is inferior as it is less effective and more costly. In the southeast quadrant, the intervention is superior, as it is more effective and less costly. In the other quadrants, the cost-effectiveness depends on the willingness to pay for a certain effect (northeast [NE]) or the willingness to accept savings for a loss in effectiveness (southwest).

The CEAC shows the probability of the intervention being cost-effective at a certain threshold value. The amount society is to pay for a certain effect. This represents the willingness-to-pay (WTP) threshold. In the Netherlands, this WTP for an improved QALY varies depending on the burden of disease, and is either €20,000, €50,000, or €80,000, with a higher accepted threshold for a higher burden of disease. As no data are available for the burden of disease of persons with profound intellectual and multiple disabilities, we have assumed a considerable burden of disease with a corresponding WTP of €50,000 [38]. The WTP threshold for a reduction in leakages, IMCs, or time spent on continence care is not known and will therefore not be displayed.

The ICER is calculated as follows: ICER=(Ci–Cc)/(Ei–Ec), where Ci is the total cost of the new intervention from societal perspective (SCC) of 12 weeks, Cc is the total cost from societal perspective of the comparator (RCC) of 12 weeks, Ei is the effect at the 12-week for SCC, and Ec is the effect at the 12-week for RCC. The ICER is calculated based on the mean difference resulting from the bootstrapped replication on the imputed datasets. Each estimated cost and effect is plotted on a CE-plane.

#### Sensitivity, Subgroup, and Scenario Analyses

Six sensitivity analyses, two subgroup analyses, and two scenario analyses were performed to assess the robustness of the findings with respect to the base-case analysis. In two sensitivity analyses, the number of weekly IMCs (SA1) and the time spent on continence care (SA2) were used as outcomes instead of leakages. Next, the prices of SCC are varied, based on the new pricing model of the supplier (expected to be implemented by the start of 2025), in two one-way sensitivity analyses per price change (SA3-SA6). In this new pricing model, the license fee per person per day for using SCC is reduced, and additional services, such as training, installation, and support, are invoiced separately as they are no longer included in the license fee. A discount based on the number of users, as well as a loyalty discount, may be applicable. First, the price of SCC is varied based on a “small organization, small implementation” setting, in which 30 persons with profound intellectual and multiple disabilities use SCC. Due to this “smaller” implementation setting, there is a small discount on the SCC license fee, and the organization chooses to purchase additional services such as training and support from the supplier. SA3 has the same outcome measures as the base case analyses (leakages and QALY), while SA4 also examines weekly IMCs. The second price variation is based on a “large organization, large implementation” setting, where SCC is used for 100 persons with profound intellectual and multiple disabilities. A larger discount is applied along with a loyalty discount, resulting in a lower day price for using SCC. Furthermore, in this setting, the organization established internal smart diaper expertise teams, thereby limiting their need for purchasing additional services (Table S1 in Multimedia Appendix 5). Again, SA5 has the same outcome measures as the base case, while SA6 focuses on weekly IMCs. Table S1 in Multimedia Appendix 5 details the implementation and the day price of using SCC.

The subgroup and scenario analyses are designed in accordance with the effectiveness study [27]. The subgroup analyses are per-protocol analyses with the same outcome measures as the base case (SUA1) and with weekly IMCs as an outcome (SUA2). The scenario analyses excluded organization C and selected those participants who used SCC according to the protocol. As the previous effectiveness study found that organization C was responsible for the unexpected outcome in the number of weekly leakages due to implementation difficulties [27]. In SCA1, the number of weekly leakages and QALY is taken as an outcome, while for SCA2, the number of weekly IMCs is used.

### Ethical Considerations

This study was approved by the Medical Ethics Committee of Radboudumc (NL72751.091.20). If the residential care facility had a research committee, it also reviewed the research proposal and approved participation. Written informed consent was obtained from the legal representatives of individuals with profound intellectual and multiple disabilities. The collected analog data were safely stored in a locked cabinet; participants’ names were removed and replaced with pseudonymized identifiers. Access to data was restricted to the researchers involved in conducting the analyses. The file containing the key, linking the pseudonymized identifier with personal details, was stored separately, with access limited to the first author and the project assistant. Participants did not receive any compensation for participating in the study. The residential care facilities received €10,000 reimbursement for research activities paid by the funder ZonMw.

## Results

### Descriptive Results

#### Sample

**Figure 1:**
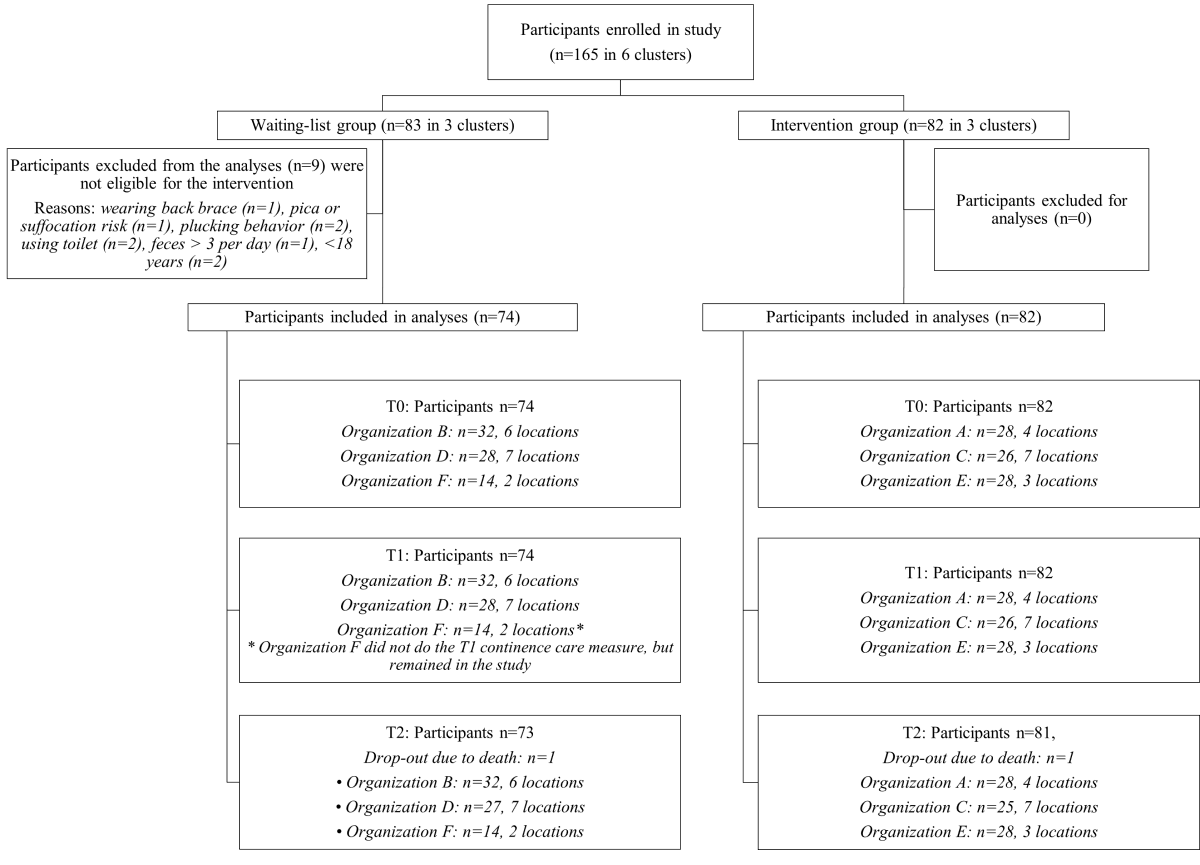
Flowchart of participants. RCC: regular continence care; SCC: smart continence care.

A total of 165 participants with profound intellectual and multiple disabilities were enrolled in the study, of which 156 were included in the intention-to-treat analyses. The RCC group consisted of 74 participants, and the SCC group consisted of 82 participants. A total of 9 participants from the RCC group were excluded from the analyses, as they were not eligible for SCC (Figure 1). Two participants died prior to the T2 measurement. In total, 23 (28%) of the 82 participants stopped receiving SCC during the 12-week study period (between T0 and T2).

The average age of the participants with profound intellectual and multiple disabilities was 48.7 (SD 14.9) years at baseline. Approximately half of the participants (70/156, 44.9%) were female. The vast majority (118/136, 86.8%) had a developmental age of <4 years. Both groups were equally distributed in terms of sex, age, and developmental age; however, they appeared to differ in terms of mobility and the presence of comorbidities. For the characteristics related to continence care, the number of leakages and weekly IMCs, there is no difference. Yet, there is a difference in the duration of an IMC, and thus, in the weekly time spent on continence care (Table 2).

**Table 2:** Baseline characteristics of the participants with profound intellectual and multiple disabilities.

Characteristics			RCC^a^ (n=74)		SCC^b^ (n=82)
**Age (years)**					
	Mean (SD)	45.4 (14.3)		51.7 (14.9)	
	Range	19-75		20-80	
**Sex, n (%)**					
	Female	35 (47.3)		35 (42.7)	
	Male	39 (52.7)		47 (57.3)	
**Developmental age (years), n (%)**					
	0-4	60 (81.2)		58 (70.7)	
	4-8	2 (2.7)		1 (1.2)	
	Unable to assess	8 (10.8)		7 (8.5)	
	Missing	4 (5.4)		16 (19.5)	
**Mobility class^c^, n (%)**					
	A	1 (1.4)		10 (12.2)	
	B	6 (8.1)		18 (22.0)	
	C	5 (6.8)		7 (8.5)	
	D	33 (44.6)		33 (40.2)	
	E	18 (24.3)		8 (9.8)	
	Missing	11 (14.9)		6 (7.3)	
**Comorbidities, n (%)**					
	Epilepsy	46 (62.2)		42 (51.2)	
	Swallow problems	40 (54.1)		32 (39.0)	
	Urine tract infections	16 (21.6)		11 (13.4)	
	Incontinence-associated dermatitis	18 (24.3)		9 (11.0)	
	Skin problems	35 (47.3)		35 (42.7)	
	Missing	0 (0)		1 (1.2)	
**Continence care^d^, mean (SD)**					
	Leakages per week	3.81 (3.03)		3.78 (2.67)	
	IMCs^e^	20.94 (6.29)		20.29 (4.28)	
	Duration in minutes per IMC	15.60 (5.63)		12.12 (7.70)	
	Weekly time spent on continence care	318.36 (127.61)		238.15 (146.31)	

^a^RCC: regular continence care.

^b^SCC: smart continence care.

^c^Mobility classes [39] were defined as follows: (A) the client can perform the task independently, with or without aids or special adaptations; (B) the client cannot perform the task independently, but the required assistance does not pose a risk of physical strain for the caregiver; (C) the client requires assistance, and the caregiver must physically support the transfer to ensure safety; (D) the client cannot perform the task independently and requires a mechanical aid, such as a lift; and (E) the client is entirely dependent on the caregiver for transfers and cannot play an active role.

^d^For continence care, n=1 missing in the RCC group.

^e^IMC: incontinence material change.

#### Observed Data: Quality of Life

At baseline, the utilities differ between the RCC and SCC groups. For the RCC group, this was, on average, 0.26 (SD 0.19), and for the SCC group, it was 0.34 (SD 0.20). At T2, the scores were 0.25 (SD 0.18) and 0.31 (SD 0.23) for the RCC and SCC groups, respectively. Using the area under the curve approach, the utility scores were 0.25 (SD 0.17) for the RCC group and 0.33 (SD 0.19) for the SCC group. The utilities were calculated for 120 participants, as they could not be generated for those with incomplete health states. As presented in Multimedia Appendix 6, the domains of pain or comfort and anxiety or depression had the highest rate of missing answers (5%-17%).

### Health Economic Results

#### Bootstrapped and Imputed Costs

Descriptive results from the bootstrapped and imputed resource use and cost variables show that caregivers’ time spent on continence care accounted for a substantial proportion (RCC: 1950/2247, 87%; SCC: 1457/2428, 60%) of the intervention costs (D; Multimedia Appendix 7). Yet, due to the baseline difference in the time spent on continence care, no conclusions can be drawn based on the displayed differences for this variable between the groups, except for their relative contribution to the intervention costs. Costs related to laundry, skin, and wound care are minor and do not exceed 5% of the total intervention costs (RCC: 54/2247, 2.4%; SCC: 76/2428, 3.1%). For the other variables in the categories “intervention costs” (D), “other health care costs” (E), and “cost for participant and family” (E), there are no noticeable or relevant differences.

Based on the adjusted results from the base case analyses, SCC is estimated to have higher total societal costs than RCC when correcting for the baseline imbalances. The total societal costs over 12 weeks are estimated to be €352 higher for SCC than for RCC, with a corresponding 95% CI of €–0.085 to €731. The total societal costs for RCC are estimated at €29,588 with a 95% CI of €29,221-€29,994, compared to €29,941 with a 95% CI of €29,670-€30,194 for the SCC group (Table 3).

**Table 3:** Outcomes of the base case, sensitivity, scenario, and subgroup analyses based on the bootstrapped (R=2000) and imputed (M=10) data, linear mixed model analyses (correcting for baseline differences)^a^.

			Number of participants				Total costs, estimated mean € (95% CI)				Effectiveness, estimated mean difference (95% CI)					Costs, estimated mean difference (95% CI)			Incremental cost-effectiveness and utility ratio		
			RCC^b^		SCC^c^		RCC		SCC		Clinical effectiveness		QALY^d^		Total societal costs			ICER^e^ (quadrant CE-plane^f^)			ICUR^g^ (quadrant CE-Plane)
**Base case**																					
	Base case	74		82		29,588.37 (29,220.61 to 29,933.95)		29,940.52 (29,699.84 to 30,194.11)		Decrease in leakages: –1.058 (–1.878 to –0.262)		0.003 (–0.032 to 0.036)		352.15 (–0.085 to 731.31)			–333 (NW^h^)			139,608 (NE^i^)	
**Sensitivity analyses**																					
	Different clinical outcomes (SA1^j^)	74		82		29,588.37 (29,220.61 to 29,933.95)		29,940.52 (29,699.84 to 30,194.11)		Decrease in IMCs^k^: 1.976 (0.733 - 3.229)		N/A^l^		352.15 (–0.085 to 731.31)			178 (NE)			N/A	
	Different clinical outcomes (SA2)	74		82		29,588.37 (29,220.61 to 29,933.95)		29,940.52 (29,699.84 to 30,194.11)		Decrease in duration of continence care per week: –3.345 (–32.108 to 25.742)		N/A		352.15 (–0.085 to 731.31)			–105 (NW)			N/A	
	Price variation: “Small organization small implementation” (SA3)	74		82		29,588.58 (29,220.69 to 29,934.54)		29,645.78 (29,405.41 to 29,898.78)		Decrease in leakages: –1.058 (–1.878 to –0.262)		0.003 (–0.032 to 0.036)		57.20 (–294.73 to 435.70)			–54 (NW)			22,6745 (NE)	
	Price variation: “Small organization small implementation” (SA4)	74		82		29,588.58 (29,220.69 to 29,934.54)		29,645.78 (29,405.41 to 29,898.78)		Decrease in IMCs: 1.976 (0.733 to 3.229)		N/A		57.20 (–294.73 to 435.70)			29 (NE)			N/A	
	Price variation: “Large organization large implementation” (SA5)	74		82		29,588.58 (29,220.69 to 29,934.54)		29,489.54 (29,249.17 to 29,742.54)		Decrease in leakages: –1.058 (–1.878 to –0.262)		0.003 (–0.032 to 0.036)		–99.04 (–450.97 to 279.46)			94 (SW^m^)			–39,265 (SE^n^)	
	Price variation: “Large organization large implementation” (SA6)	74		82		29,588.58 (29,220.69 to 29,934.54)		29,489.54 (29,249.17 to 29,742.54)		Decrease in IMC: 1.976 (0.733 to 3.229)		N/A		–99.04 (–450.97 to 279.46)			–50 (SE)			N/A	
**Subgroup analyses**																					
	Per protocol (SUA1^o^)	73		59		29,621.48 (29,203.91 to 29,999.88)		29,978.33 (29,725.61 to 30,267.66)		Decrease in leakages: –1.021 (–1.970 to –0.062)		0.007 (–0.024 to 0.040)		356.86 (–43.76 to 774.50)			–349 (NW)			48,236 (NE)	
	Per protocol, (SUA2)	73		59		29,621.48 (29,203.91 to 29,999.88)		29,978.33 (29,725.61 to 30,267.66)		Decrease in IMCs: 2.020 (0.679 to 3.399)		N/A		356.86 (–43.76 to 774.50)			177 (NE)			N/A	
**Scenario analyses**																					
	Excluding organization C, per protocol (SCA1^p^)	73		40		29,607.36 (29,210,57 to 30,002.98)		30,077.46 (29,672.12 to 30,510.13)		Decrease in leakages: –0.276 (–1.291 to 0.755)		–0.005 (–0.046 to 0.035)		470.10 (–5.10 to 968.11)			–1703 (NW)			–98,657 (NW)	
	Excluding organization C, per protocol (SCA2)	73		40		29,607.36 (29,210,57 to 30,002.98)		30,077.46 (29,672.12 to 30,510.13)		Decrease in IMCs: 2.431 (0.928 to 3.918)		N/A		470.10 (–5.10 to 968.11)			193 (NW)			N/A	

^a^All costs are reported in euros. Conversion rate of €1=US $1.05469 (as of December 6, 2024).

^b^RCC: regular continence care.

^c^SCC: smart continence care.

^d^QALY: Quality Adjusted Life Years.

^e^ICER: incremental cost-effectiveness ratio plane.

^f^CE-plane: cost-effectiveness plane.

^g^ICUR: incremental cost-utility ratio.

^h^NW: northwest quadrant in the CE-plane representing less effective, more costly.

^i^NE: northeast quadrant in the CE-plane representing more effective, more costly.

^j^SA: subgroup analysis.

^k^IMC: incontinence material change.

^l^Not applicable.

^m^SW: southwest quadrant in the CE-plane representing less effective, less costly.

^n^SE: southeast quadrant in the CE-plane representing more effective, less costly.

^o^SUA: subgroup analysis.

^p^SCA: scenario analysis.

#### Clinical Outcome and Quality of Life

Based on the adjusted results from the base case analyses, SCC is estimated to be ineffective in decreasing the number of leakages. The estimated mean difference for decreasing leakages is –1.058 (95% CI –1.878 to –0.262), representing an increase in the number of leakages within the SCC group compared to the group using RCC (Table 3). No improvements in quality of life were found when correcting for the imbalances in utility at baseline. The estimated mean difference of the corrected imputed and bootstrapped data gives an estimate of 0.003 (95% CI –0.032 to 0.036; Table 3).

#### Base Case: Cost-Effectiveness Analysis

**Figure 2:**
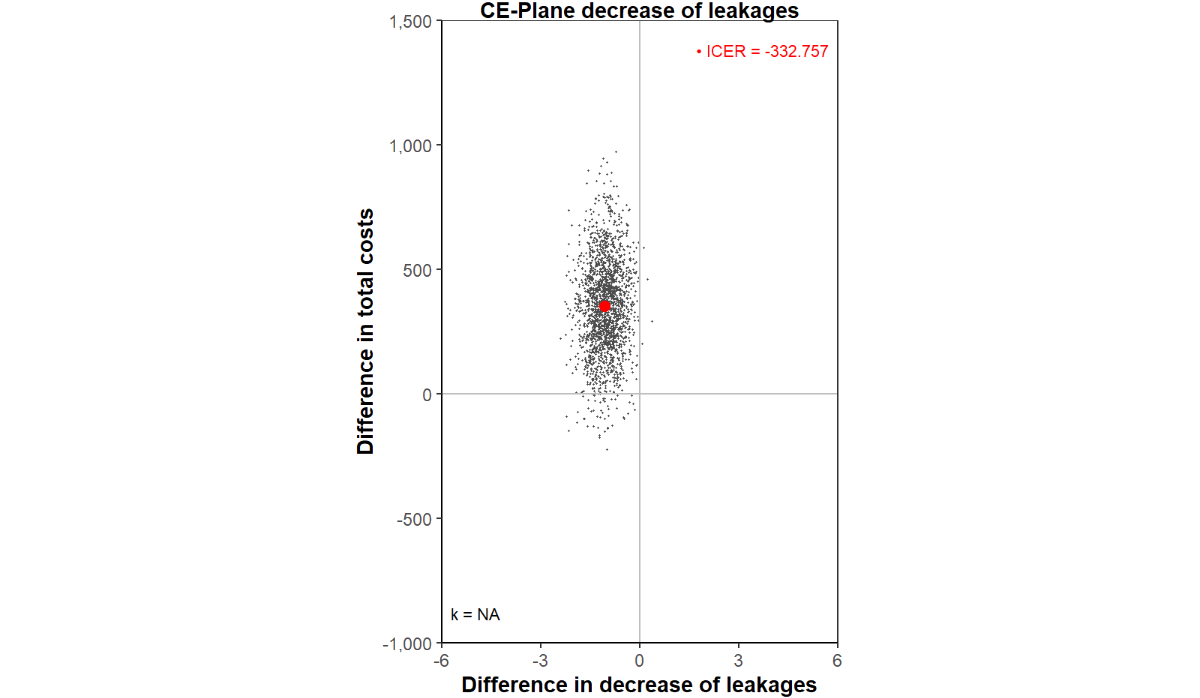
CE-plane for the base case analysis. All costs are reported in euros. Applied conversion rate of EUR €1=US $1.05469 (as of December 6, 2024). CE-plane: cost-effectiveness plane; ICER: incremental cost-effectiveness ratio.

According to the health economic results from the base case cost-effectiveness analysis, from a societal perspective with leakages as an outcome, SCC is associated with a higher chance of being more expensive (€352) and less effective compared to RCC, as it does not decrease the number of weekly leakages (–1.058). This leads to an estimated inferior ICER of –333 (Table 3), that is, an additional societal cost of €333 per 12 weeks, resulting in one extra leakage per week. These results are shown in the CE-plane (Figure 2), where most bootstrap replications fall within the NW quadrant (higher costs, less effect).

#### Base Case: CUA

**Figure 3:**
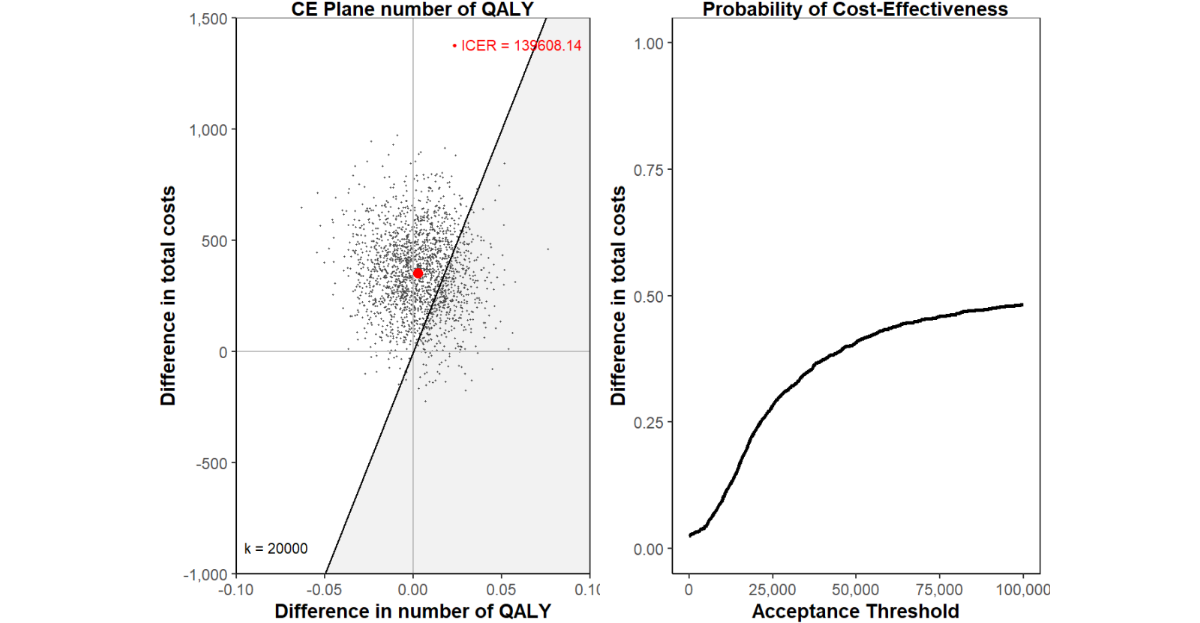
CE-plane and acceptability curve of the base-case analysis QALY as utility outcome. All costs are reported in euros. Applied conversion rate of EUR €1=US $1.05469 (as of December 6, 2024). CE-plane: cost-effectiveness plane; QALY: Quality Adjusted Life Years.

For the base case CUA, the corrected imputed and bootstrapped results suggest a relatively large uncertainty in the difference in QALYs between the two groups, with an estimated mean difference of 0.003 (95% CI –0.032 to 0.036; Table 3). In the CE-plane, this is reflected by bootstrapped replications falling between the NW and NE quadrants (Figure 3). As shown in the CEAC plot, the probability of cost-effectiveness for SCC compared to RCC is estimated to be between 0 and 0.5 for the WTP threshold range of €0-€100,000. At the WTP threshold of €50,000 cost per QALY gained, a 40% chance that the SCC is cost-effective is estimated.

#### Sensitivity Analyses

Based on the health economic results from the additional analyses, when the weekly number of IMCs is taken as an outcome (SA1), most bootstrapped replications lie in the NE quadrant of the CE-plane, indicating a positive effect (ie, a decrease in the number of weekly IMCs) with higher societal costs on average. When the time spent on continence care is considered as an outcome (SA2), results show a large uncertainty related to the effectiveness in decreasing time spent on continence care, with most bootstrapped points lying in both northern quadrants, and with the ICER being located in the NW quadrant (more costly, less effective; Table S3 and Figures S1 and S12 in Multimedia Appendix 5).

In four additional sensitivity analyses (SA3-SA6), in which the prices were based on the new pricing model of the supplier, the effect on the clinical outcomes remained the same, but with lower total societal costs (base case mean difference: €352). For the “small organization, small implementation” setting (SA3-SA4), the mean difference in societal costs is €57, but with a large uncertainty as indicated by the 95% CI interval of €–295 to €436. This is represented in terms of results on CE-planes by bootstrapped estimates that are spread between the north and south quadrants. For the “large organization, large implementation” setting (SA5-SA6), the estimated mean difference of the total societal costs is €–99, with a 95% CI of €–451 to €279, with bootstrapped estimates in the south quadrant, but with substantial uncertainty about whether SCC is cheaper then RCC (Table S3 and Figures S3, S4, S8, S9, S13, and S14 in Multimedia Appendix 5). For the CUA, we observe a slight increase in the chance of being cost-effective, increasing from approximately 40% to 50% and 60% for “small organization, small implementation” setting and “large organization, large implementation” setting, respectively (Table S3 and Figures S7-S9 in Multimedia Appendix 5).

#### Subgroup Analyses

From a societal perspective, the results of the subgroup per-protocol analyses (SUA1 and SUA2) align with those of the base case analyses (Table S3 and Figures S5, S10, and S15 in Multimedia Appendix 5). In the per-protocol analyses, 23 of the 82 (28%) participants stopped SCC before 12 weeks within the intervention group. Reasons included caregivers’ dissatisfaction with the IM absorptive capacity and fit of the IM, difficulties in changing the work routines, the occurrence of skin problems, participants who did not accept the clip, or the voiding of large amounts of urine that could not be contained by any IM. One participant passed away in the intervention group. In the waiting-list group, one participant (1/74, 1.4%) did not follow the per-protocol as this participant passed away. More details and the percentage of occurrence of these reasons can be found elsewhere [27].

#### Scenario Analyses

**Figure 4:**
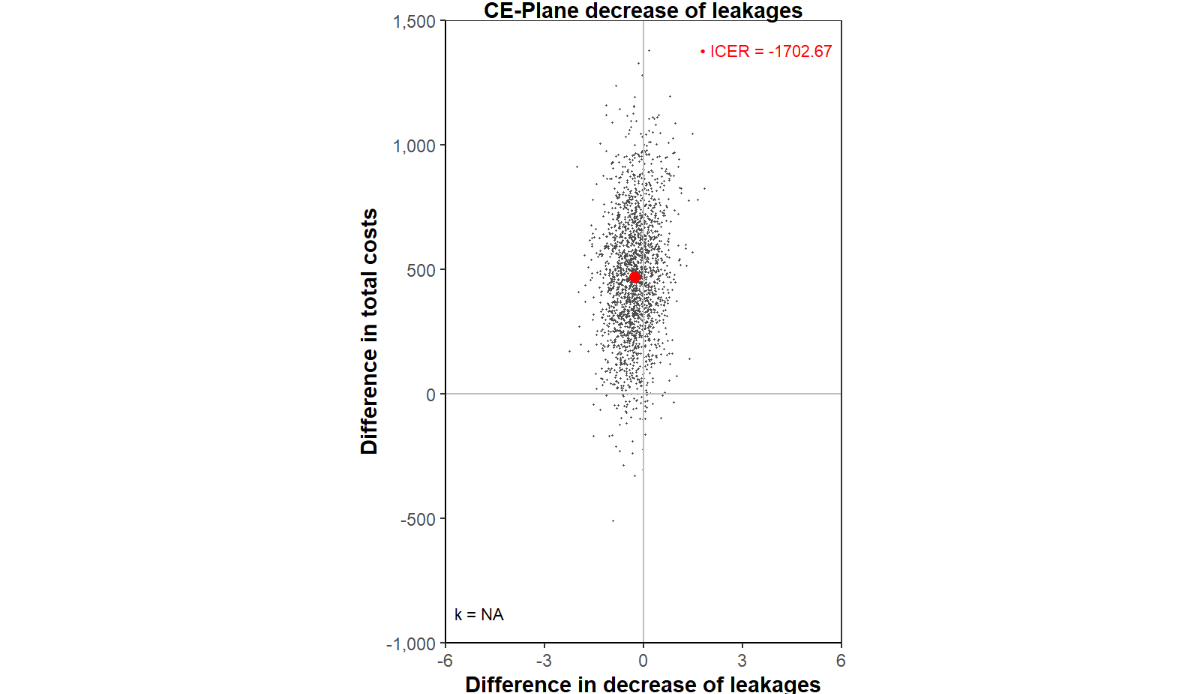
CE-plane for the scenario analysis (per-protocol and excluding organization C) for the decrease in leakages. All costs are reported in euros. Applied conversion rate of EUR €1=US $1.05469 (as of December 6, 2024). CE-plane: cost-effectiveness plane; ICER: incremental cost-effectiveness ratio.

The per-protocol scenario analyses excluding organization C (SCA1) showed that one organization (organization C) impacted the clinical outcome, the number of weekly leakages, and thereby influenced the overall probability of this outcome being inferior. This is reflected in the economic results shown on the CE-plane, which shows bootstrapped replications located in both the NE and NW quadrants (SCA1; Figure 4), compared to the base case, where these are in the NE (inferior) quadrant (Table 3). On outcome utility (SCA1) and weekly IMCs (SUA2), results in the scenario analyses are quite similar to the intention-to-treat analyses of the base case (Table S3 and Figures S11 and S16 in Multimedia Appendix 5).

## Discussion

### Principal Findings

The need for an economic evaluation of SCC for persons with profound intellectual and multiple disabilities is evident, considering the potential benefit SCC can bring to persons with profound intellectual and multiple disabilities and their caregivers, the decreasing number of caregivers, and the increasing demand for health care services in current times [16]. This study is the first to evaluate the cost-effectiveness and cost-utility of SCC for persons with profound intellectual and multiple disabilities living in residential care from a societal perspective. The base case cost-effectiveness analyses, focusing on the number of leakages, indicated that it is likely that SCC induces higher societal costs than RCC. In line with our effect study [27], this health economic evaluation also showed the unexpected reduction in leakages for the RCC group compared to the SCC group. The sensitivity and subgroup analyses confirmed the base case findings, except for the scenario analysis, which excluded one organization from the intervention group. In this case, no difference in the reduction of weekly leakages was found between the two groups (SCC vs RCC), meaning that the aforementioned difference was due to the higher number of leakages in this specific organization. van Cooten et al [27] argued that this was a consequence of reported difficulties with implementation and high staff turnover, demonstrating that the implementation process is complex and that achieving the potential benefits is not self-evident. Still, however, this did not alter the conclusion that SCC is unlikely to be cost-effective in relation to reducing the weekly number of leakages.

The base case CUA reported an equal likelihood of higher societal costs and showed substantial uncertainty around the quality of life. Sensitivity analyses in which the pricing of SCC was varied showed that with the new pricing model, especially if the care organization opts for a large implementation and qualifies for an additional discount, the likelihood of achieving cost savings from a societal perspective increase. This leads to an increment in the likelihood that SCC is, on average, cheaper than RCC, with the incremental cost-utility ratio mainly falling below the acceptable WTP threshold of €50,000.

Analyses regarding the number of weekly IMCs as outcome measures revealed somewhat more promising results. Sensitivity analyses showed that the number of IMCs decreased effectively when using SCC compared to RCC, which is in line with the previous findings on effectiveness reported by van Cooten et al [27]. This finding is further supported by the subgroup and scenario analyses. Decreasing the number of IMCs implies that persons with profound intellectual and multiple disabilities experience fewer disadvantages of unnecessary IMCs. However, the analyses indicate that SCC is not necessarily a time-saving intervention, as no effect was found regarding time spent on continence care between the two groups.

### Strengths and Limitations

This study has several strengths, including its robust CRT design, leading to a high internal validity of the study findings. Furthermore, we adhered to the Dutch guidelines for EEH and CHEERS 2022 reporting guidelines [17,28,29]. State-of-the-art statistical analyses were performed using linear mixed regression methods, considering the clustering of the data, accounting for missing data under the missing at random assumption via multiple imputation, and correcting for important baseline imbalances at baseline for both costs and outcomes. These analyses were conducted by an independent statistician (AG) assisted by a researcher (VJCC). Correcting for baseline imbalances, particularly in the time spent on continence care, was highly relevant because staff costs—one of the main cost drivers of the intervention costs—varied substantially between the groups. Neglecting this imbalance could have led to false conclusions. Additionally, the study benefited from a large sample size, particularly in the research field for persons with profound intellectual and multiple disabilities. In this field, trials are rare and sample sizes are mainly below 10 participants [11,40,41]. Only two participants dropped out as they deceased. Various disability care organizations across the Netherlands are included, which contributes to the generalizability of our results.

To our knowledge, this is the first economic evaluation of an intervention using care technology targeted at individuals with profound intellectual and multiple disabilities. Besides knowledge on the cost-effectiveness of SCC, it also provided us with a deeper understanding of the complexities involved in conducting health economic analyses in the residential care sector, illustrated by several limitations of this study. First, the time horizon of our study was limited to a time frame of 12 weeks. As a result, we did not include and amortize the start-up costs, which would have resulted in a more accurate estimate of the total societal costs for the SCC group. Second, some care teams in the intervention group might still be learning how to use the SCC technology, and thus, the measured effectiveness might improve when more time is allowed. Third, due to time constraints, medication costs were not included, leading to incomplete societal costs. Yet, the lack of these costs is not expected to affect our conclusions. It is unlikely that medication regimes, and thus total medication costs, would change within 12 weeks due to the type of continence care received.

The third and most important limitation concerns the use of the EQ-5D-5L. Although EQ-5D-5L is considered more discriminating in patients with more severe illnesses than the Short-Form Six Dimensions [42] and is designated as the default option in health economic evaluations and obligatory according to the Dutch guideline for EEH [3], we experienced its use as inappropriate and even problematic for individuals with profound intellectual and multiple disabilities. Versteegh et al [43] state that “EQ-5D-5L scores have to be presented in the reference case (base case), even when they might be considered inappropriate.” Even though Versteegh et al [43] recognize its limited value beyond the cure sector, they are clear about its obligatory use. Present alternatives alongside the EQ-5D-5L scores are allowed, but unfortunately, a validated health utility instrument for persons with profound intellectual and multiple disabilities or even persons with intellectual disabilities in general is lacking [44]. Given the severe disabilities experienced by individuals with profound intellectual and multiple disabilities, no improvement can be expected in most domains of the EQ-5D-5L. In particular, the domains of mobility and self-care were most inappropriate for this population. To our knowledge, the suitability of EQ-5D-5L for individuals with profound intellectual and multiple disabilities has not been studied. A literature search on using the EQ-5D-5L as an instrument for health utility only revealed one protocol paper focused on individuals with profound intellectual and multiple disabilities [45]. We recommend that future CUA on interventions for individuals with profound intellectual and multiple disabilities consider developing a more suitable instrument for this group, as also acknowledged by Benedetto et al [45].

### Comparison With Prior Work and Future Research

In our search for evidence on the cost-effectiveness of SCC, we only found a limited number of studies. The studies we did find focused on the use of SCC in older people’s care, which limits the comparability with this study.

Abraham et al [25] developed a de novo cost-effectiveness economic model to estimate the cost-utility of an SCC system for older nursing home residents. This system contains 72-hour monitoring of the voiding pattern, which caregivers use to construct a personalized continence care plan. They found this solution to be cost-effective, as most of their model outcomes showed an increase in quality of life and a decrease in costs from a health care provider perspective. Their model considered the evolving care needs of older residents due to changing health states and considered that these individuals typically stay in residential care for a relatively short duration (2.34 y on average). They found a CAD $1467 (US $1038, with a conversion rate of CAD $1=US $0.7074, as of December 6, 2024) saving per resident and recommended further implementation due to this positive financial and clinical impact. However, it is important to note that this model relies on various cost and effectiveness assumptions derived from different literature sources and adopts a health care provider perspective, which differs from the societal perspective used in this study.

Three other studies focused on the health care provider perspective. A Canadian study, which examined the effectiveness of a personalized continence care plan developed with electronic monitoring, reported a significant cost reduction in the materials used for the night [46]. However, they did not compare these cost savings to the additional costs for the monitoring system or the other relevant cost aspects, such as staff costs. Contrary to our results, two Dutch studies [24,47] reported a time saving of 0.05-0.07 full-time equivalents in older people’s care per resident using the same SCC solution as in this study. These results, however, are based on a single-arm design.

Future economic evaluation studies on SCC for persons with profound intellectual and multiple disabilities in residential disability care should include the health care provider perspective. This study focuses on the economic evaluation of SCC from a societal perspective. However, especially in current times, care organizations need to develop their own “business case” to decide whether or not to implement SCC. For care providers, knowledge of intervention costs in relation to the benefits for their residents is particularly relevant. Additionally, it would be valuable to investigate whether implementing SCC can lead to time savings and a decrease in the number of weekly leakages over a longer period. Most importantly, future studies should invest in developing a suitable instrument for measuring quality of life in persons with profound intellectual and multiple disabilities, using input from a multidisciplinary expert group, to be used in cost-effectiveness and cost-utility studies for this specific group.

### Conclusions

This study evaluated the cost-effectiveness and cost-utility of SCC versus RCC for persons with profound intellectual and multiple disabilities in the Netherlands from a societal perspective. The results of this economic evaluation are not conclusive. Although the total societal costs are likely to be higher for SCC, we cannot simply state that SCC is inferior compared to RCC. It was found that SCC, although not effective in reducing the number of leakages, is effective in reducing the number of weekly IMCs. This reduction implies a decrease in the number of unnecessary changes, thereby decreasing the frequency with which persons with profound intellectual and multiple disabilities are interrupted in their daily activities. However, this has not translated into time savings for staff when comparing the differences between the groups. The impact of SCC on the quality of life of persons with profound intellectual and multiple disabilities appears to be mixed. Caution is warranted when drawing conclusions on quality of life due to, on the one hand, the small observed effects, and on the other hand, the inappropriateness of the EQ-5D-5L instrument for persons with profound intellectual and multiple disabilities. We found that proper implementation is crucial and has impacted whether or not the advantages of the use of SCC are reached.

One last important note is that the use of technologies such as SCC should not be based solely on cost-effectiveness and cost-utility outcomes from a societal perspective. This technology has the potential to offer value by generating data that can support personalized care, but the actual realization of this added value may vary among individuals.

## Appendix

Multimedia Appendix 1 CHEERS: consolidated health economic evaluation reporting standards.

Multimedia Appendix 2 CONSORT-EHEALTH (V 1.6.1) checklist.

Multimedia Appendix 3 Cost calculations.

Multimedia Appendix 4 Technical details on the health economic analyses.

Multimedia Appendix 5 Sensitivity, subgroup and scenario analyses.

Multimedia Appendix 6 EQ-5D domain scores.

Multimedia Appendix 7 Imputed and bootstrapped resource use and costs per variable.

## Abbreviations

CEAC: cost-effectiveness acceptability curve
CE-plane: cost-effectiveness plane
CHEERS: Consolidated Health Economic Evaluation Reporting Standards
CONSORT-EHEALTH: Consolidated Standards of Reporting Trials of Electronic and Mobile Health Applications and Online Telehealth
CRT: cluster-randomized trial
CUA: cost-utility analyses
DIRUM: Database for Instruments of Resource Use Measurements
EEH: Economic Evaluations in Healthcare
ICER: incremental cost-effectiveness ratio
IM: incontinence material
IMC: incontinence material change
iMCQ: iMTA Medical Cost Consumption Questionnaire
NE: northeast
NW: northwest
QALY: Quality Adjusted Life Years
RCC: regular continence care
SCC: smart continence care
WTP: willingness-to-pay

## Data Availability

The datasets generated or analyzed during this study are not publicly available due to the sensitive nature of the participants, and informed consent was limited to the specific purpose of this study, but are available from the corresponding author on reasonable request.

## References

[1] Nair R, Chen M, Dutt A, Hagopian L, Singh A, Du M (2022). Significant regional inequalities in the prevalence of intellectual disability and trends from 1990 to 2019: a systematic analysis of GBD 2019. Epidemiol Psychiatr Sci.

[2] (2024). Global Burden of Disease (2024) with major processing by our world in data. IHME.

[3] McKenzie K, Milton M, Smith G, Ouellette-Kuntz H (2016). Systematic review of the prevalence and incidence of intellectual disabilities: current trends and issues. Curr Dev Disord Rep.

[4] Maulik PK, Mascarenhas MN, Mathers CD, Dua T, Saxena S (2011). Prevalence of intellectual disability: a meta-analysis of population-based studies. Res Dev Disabil.

[5] Woittiez I, Eggink E, Ras M (2019). Het aantal mensen met een licht verstandelijke beperking: een schatting [The number of people with mild intellectual disabilities: an estimate]. Sociaal en Cultureel Planbureau.

[6] (2019). Feiten en cijfers in de gehandicaptenzorg [Facts and figures in disability care]. VGN.

[7] Vugteveen J, van der Putten AAJ, Vlaskamp C (2014). Inventarisatieonderzoek Mensen met Ernstige Meervoudige Beperkingen: Prevalentie en Karakteristieken [Inventory Study of People with Severe Multiple Disabilities: Prevalence and Characteristics].

[8] Nakken H, Vlaskamp C (2007). A need for a taxonomy for profound intellectual and multiple disabilities. J Policy Pract Intellect Disabil.

[9] (2024). Uitgaven gezondheids- en welzijnszorg; zorgtypes en financiering [Health and welfare expenditure; types of care and financing]. CBS.

[10] Beeckman D (2017). A decade of research on incontinence-associated dermatitis (IAD): evidence, knowledge gaps and next steps. J Tissue Viability.

[11] Piekema L, Ten Brug A, Waninge A, van der Putten A (2024). From assistive to inclusive? A systematic review of the uses and effects of technology to support people with pervasive support needs. J Appl Res Intellect Disabil.

[12] Vázquez A, Jenaro C, Flores N, Bagnato MJ, Pérez MC, Cruz M (2018). E-health interventions for adult and aging population with intellectual disability: a review. Front Psychol.

[13] Frielink N, Oudshoorn CEM, Embregts PJCM (2021). eHealth in support for daily functioning of people with intellectual disability: views of service users, relatives, and professionals on both its advantages and disadvantages and its facilitating and impeding factors. J Intellect Dev Disabil.

[14] Bierhof I, van der Poel A (2024). Welke technologie gebruiken organisaties in de gehandicaptenzorg bij de ondersteuning van hun cliënten en medewerkers [What technology do organizations in disability care use in the care and support of their clients and employees?]. Academy Het Dorp.

[15] UnitedNations (2006). International Human Rights Law Documents.

[16] (2023). Arbeidsmarkt gehandicaptenzorgn situatieschets [Disability care labor market: a situation sketch]. VGN.

[17] (2024). Guideline for economic evaluations in healhtcare 2024. Zorginstituut Nederland.

[18] Drummond ME, Sculpher MJ, Torrance GW, O'Brien B, Stoddart G (2005). Methods for the Economic Evaluation of Health Care Programmes.

[19] (2020). Als bepalen van het verschoningsmoment een uitdaging is, maakt TENA het makkelijker [“If determining when to change your diaper is a challenge, TENA makes it easier”]. TENA.

[20] NA (2018). Abena nova with mediSens. ABENA.

[21] (2024). Wear and care the future of incontinence care. Wear&Care Technologies.

[22] (2024). Wat is WeSense. Mediq.

[23] (2020). Abena nova with medisens, snel aan de slag [Abena nova with medisens, get started quickly]. Abena.

[24] Nap HH, Suijkerbuijk S, Bierhoff I, van de Wiel R, Buimer H (2020). Onderzoeksrapportage slim incontinentiemateriaal [Research report on smart incontinence materials]. Anders werken in de zorg.

[25] Abraham K, Kanters TA, Wagg AS, Huige N, Hutt E, Al MJ (2024). Benefits of a digital health technology for older nursing home residents. A de-novo cost-effectiveness model for digital health technologies to aid in the assessment of toileting and containment care needs. PLoS One.

[26] van Cooten VJC, Gielissen MFM, van Mastrigt GAPG, den Hollander W, Evers SMAA, Smeets O, Smit F, Boon B (2022). Smart continence care for people with profound intellectual and multiple disabilities: protocol for a cluster randomized trial and trial-based economic evaluation. JMIR Res Protoc.

[27] van Cooten VJ, Gielissen MF, den Hollander W, van Mastrigt GA, Smeets O, Bongers IM, Boon B (2025). Effectiveness of smart continence care for people with profound intellectual and multiple disabilities: cluster randomized trial. J Med Internet Res.

[28] Husereau D, Drummond M, Augustovski F, de Bekker-Grob E, Briggs AH, Carswell C, Caulley L, Chaiyakunapruk N, Greenberg D, Loder E, Mauskopf J, Mullins CD, Petrou S, Pwu R, Staniszewska S (2022). Consolidated health economic evaluation reporting standards (CHEERS) 2022 explanation and elaboration: a report of the ISPOR CHEERS II good practices task force. Value Health.

[29] Husereau D, Drummond M, Augustovski F, de Bekker-Grob E, Briggs A, Carswell C, Caulley L, Chaiyakunapruk N, Greenberg D, Loder E, Mauskopf J, Mullins CD, Petrou S, Pwu RF, Staniszewska S (2022). Consolidated health economic evaluation reporting standards 2022 (CHEERS 2022) statement: updated reporting guidance for health economic evaluations. Value Health.

[30] van Cooten V, Boon B, Gielissen M, Bongers I, van Mastrigt G, Smeets O (2025). Smart personalized continence care for people with profound intellectual and multiple disabilities: a theory and practice‐ based implementation guideline for a digital innovation. J Policy Pract Intellect Disabil.

[31] (2019). EQ-5D-5L user guide. Foundation ER.

[32] Versteegh MM, Vermeulen KM, Evers SMAA, de Wit GA, Prenger R, Stolk EA (2016). Dutch tariff for the five-level version of EQ-5D. Value Health.

[33] Brooks R (1996). EuroQol: the current state of play. Health Policy.

[34] Herdman M, Gudex C, Lloyd A, Janssen M, Kind P, Parkin D, Bonsel G, Badia X (2011). Development and preliminary testing of the new five-level version of EQ-5D (EQ-5D-5L). Qual Life Res.

[35] (2018). Manual iMTA medical cost questionnaire (iMCQ). iMTA Productivity and Health Research Group.

[36] van Buuren S (2018). Flexible Imputation of Missing Data.

[37] Little RJA (1988). Missing-data adjustments in large surveys. J Bus Econ Stat.

[38] (2024). Beoordelingskader kosteneffectiviteit van zorg [Assessment framework for the cost-effectiveness of care]. Ministerie van Volksgezondheid WeS.

[39] Knibbe J, Hulshof N, Stoop AP, Friele RD (1998). Kleine hulpmiddelen: hulp voor bewoners en zorgverleners?: een verkenning naar de mogelijkheden om met kleine hulpmiddelen de zelfstandigheid van ouderen in verzorgingshuizen te bevorderen en het werk van verzorgenden lichter te maken [Small aids: help for residents and caregivers? An exploration of the possibilities of using small aids to promote the independence of elderly people in nursing homes and to make the work of caregivers easier]. Utrecht: NIVEL.

[40] Maes B, Lambrechts G, Hostyn I, Petry K (2007). Quality-enhancing interventions for people with profound intellectual and multiple disabilities: a review of the empirical research literature. J Intellect Dev Disabil.

[41] Maes B, Nijs S, Vandesande S, Van Keer I, Arthur-Kelly M, Dind J, Goldbart J, Petitpierre G, Van der Putten A (2021). Looking back, looking forward: methodological challenges and future directions in research on persons with profound intellectual and multiple disabilities. J Appl Res Intellect Disabil.

[42] Brazier J, Roberts J, Tsuchiya A, Busschbach J (2004). A comparison of the EQ-5D and SF-6D across seven patient groups. Health Econ.

[43] Versteegh M, Knies S, Brouwer W (2016). From good to better: new Dutch guidelines for economic evaluations in healthcare. Pharmacoeconomics.

[44] Benedetto V, Filipe L, Harris C, Spencer J, Hickson C, Clegg A (2023). Analytical frameworks and outcome measures in economic evaluations of digital health interventions: a methodological systematic review. Med Decis Making.

[45] Lundqvist L, Matérne M, Granberg A, Frank A, Arvidsson P, Duberg A (2020). Structured water dance intervention (SWAN) for adults with profound intellectual and multiple disabilities: study protocol. Heliyon.

[46] Rajabali SN, Hunter KF, Asaana P, McCreary ML, Nazari S, Wagg AS (2023). Effectiveness of a smart urinary continence care assessment system for nursing home residents: a quasi-experimental, sequential quantitative-qualitative methods trial. J Wound Ostomy Continence Nurs.

[47] Bouman G, Lapajian I (2019). Implementatie slim incontinentiemateriaal [Implementation of smart incontinence materials]. Eindverslag.

