# Supplementary material for: Smart Continence Care for People With Profound Intellectual and Multiple Disabilities Within Dutch Residential Care Facilities: Economic Evaluation Alongside a Cluster Randomized Trial
Source: J Med Internet Res. 2025 Oct 10;27:e72017. doi: 10.2196/72017 (PMC12552815; doi:10.2196/72017)
Supplement: Multimedia Appendix 1 [file jmir_v27i1e72017_app1.pdf]

CHEERS guideline by ‘Smart continence care for people with profound intellectual and multiple disabilities within Dutch residential care facilities: economic evaluation alongside a cluster randomized trial’ by van Cooten et al, 2025

Cheers item	Guidance for reporting	Reported in section
**1**	Title: Identify the study as an economic evaluation and specify the interventions being compared.	Title
**Abstract**		
**2**	Abstract: Provide a structured summary that highlights context, key methods, results, and alternative analyses.	Abstract
**Introduction**		
**3**	Background: Give the context for the study, the study question, and its practical relevance for decision making in policy or practice.	Background
**Methods**		
**4**	Health economic analysis plan: Indicate whether a health economic analysis plan was developed and where available.	Method>Study design, here referred to per protocol study which.
**5**	Study population: Describe characteristics of the study population (such as age range, demographics, socioeconomic, or clinical characteristics).	Table 1, also see in and exclusion criteria under Method>participants’ recruitment
**6**	Setting and location: Provide relevant contextual information that may influence findings.	Method>participants’ recruitment
**7**	Comparators: Describe the interventions or strategies being compared and why chosen.	Method>procedure>regular care
**8**	Perspective: State the perspective(s) adopted by the study and why chosen.	Introduction
**9**	Time horizon: State the time horizon for the study and why appropriate.	Method>study design
**10**	Discount rate: Report the discount rate(s) and reason chosen.	Method>valuation
**11**	Selection of outcomes: Describe what outcomes were used as the measure(s) of benefit(s) and harm(s).	Method>clinical outcome & quality of life and section: costs>identification and valuation
**12**	Measurements of outcomes: Describe how outcomes used to capture benefit(s) and harm(s) were measured.	Method>clinical outcome & quality of life
**13**	Valuation of outcomes: Describe the population and methods used to measure and value outcomes.	Method>clinical outcome & quality of life
**14**	Measurement and valuation of resources and costs: describe how costs were valued	Method>Costs: identification, measurement and valuation>valuation
**15**	Currency, price date, and conversion: Report the dates of the estimated resource quantities and unit costs, plus the currency and year of conversion.	Method>Costs: identification, measurement and valuation>valuation

**16**	Rationale and description of model: If modeling is used, describe in detail and why used. Report if the model is publicly available and where it can be accessed.	Not applicable
**17**	Analytics and assumptions: Describe any methods for analysing or statistically transforming data, any extrapolation methods, and approaches for validating any model used.	Health economic analyses and supplementary file 2
**18**	Characterizing heterogeneity: Describe any methods used for estimating how the results of the study vary for subgroups.	Method>Health economic analyses> Sensitivity, subgroup and scenario analyses
**19**	Characterizing distributional effects: Describe how impacts are distributed across different individuals or adjustments made to reflect priority populations.	Not applicable
**20**	Characterizing uncertainty: Describe methods to characterize any sources of uncertainty in the analysis.	Method>Health economic analyses> Sensitivity, subgroup and scenario analyses
**21**	Approach to engagement with patients and others affected by the study: Describe any approaches to engage patients or service recipients, the general public, communities, or stakeholders (such as clinicians or payers) in the design of the study.	Method>study design, refers to session with parents of persons with PIMD in collaboration with a patient organization. Method>study design also describes the brainstorming session with care professionals
**Results**		
**22**	Study parameters: Report all analytic inputs (such as values, ranges, references) including uncertainty or distributional assumptions.	See table 3 and supplementary file 4
**23**	Summary of main results: Report the mean values for the main categories of costs and outcomes of interest and summarize them in the most appropriate overall measure.	See table 3 and Results>Health economic results
**24**	Effect of uncertainty: Describe how uncertainty about analytic judgments, inputs, or projections affect findings. Report the effect of choice of discount rate and time horizon, if applicable.	Results>Sensitivity analyses and Results>Subgroup and scenario analyses
**25**	Effect of engagement with patients and others affected by the study: Report on any difference patient/service recipient, general public, community, or stakeholder involvement made to the approach or findings of the study	Methods>Study design
**Discussion**		
**26**	Study findings, limitations, generalizability, and current knowledge: Report key findings, limitations, ethical or equity considerations not captured, and how these could affect patients, policy, or practice.	Discussion

**Other relevant items**		
**27**	Source of funding: Describe how the study was funded and any role of the funder in the identification, design, conduct, and reporting of the analysis	Funding
**28**	Conflicts of interest: Report authors conflicts of interest according to journal or International Committee of Medical Journal Editors requirements.	Conflict of interest

1. Husereau D, Drummond M, Augustovski F, de Bekker-Grob E, Briggs AH, Carswell C, et al. Consolidated Health Economic Evaluation Reporting Standards 2022 (CHEERS 2022) Statement: Updated Reporting Guidance for Health Economic Evaluations. Value in Health. 2022 2022/01/01/;25(1):3-9. doi: https://doi.org/10.1016/j.jval.2021.11.1351.
