# Supplementary material for: Smart Continence Care for People With Profound Intellectual and Multiple Disabilities Within Dutch Residential Care Facilities: Economic Evaluation Alongside a Cluster Randomized Trial
Source: J Med Internet Res. 2025 Oct 10;27:e72017. doi: 10.2196/72017 (PMC12552815; doi:10.2196/72017)
Supplement: Multimedia Appendix 3 [file jmir_v27i1e72017_app3.docx]

This supplementary file presents the valuation of each cost item if not provided by the Dutch guideline for economic evaluations in health (EEH)[1]. The prices are summed in Table 1. All prices are displayed in EUROS. To convert to US $, use the current conversion rate of EUR €1=US $1.05469 (as per December 6, 2024).

Table S1: Each variable and its unit price. All costs are presented in EUROS, to convert to US $. Conversion rate: EUR €1=US $1.05469 (as per December 6, 2024).

| **Product** | **Applicable for type of continence care** | | **Price (2022) € incl. taxes** | **Unit** |
| --- | --- | --- | --- | --- |
|  | **SCC** | **RCC** |  |  |
| **Intervention costs for continence care** | | | | |
| Staff providing continence care | Yes | Yes | € 36.74 | Per hour |
| Smart incontinence material | Yes | No | € 1.30 | Per incontinence material change |
| Regular incontinence material | No | Yes | € 0.74 | Per incontinence material change |
| Disposables | Yes | Yes | € 0.37 | Per incontinence material change |
| Wound and skin care, during continence care | Yes | Yes | € 0.73 | Per event |
| Laundry full bed | Yes | Yes | € 2.58 | Per event |
| Laundry half bed | Yes | Yes | € 1.29 | Per event |
| Laundry set of clothes | Yes | Yes | € 0.86 | Per event |
| License fee | Yes | No | € 6.05 | Per day |
| Relays | Yes | No | € 0.56 | Per day |
| Clip | Yes | No | € 0.22 | Per day |
| **Other healthcare costs** | | | | |
| Stay in disability care, excluding continence care, including the activity center | Yes | Yes | € 319.78 | Per day |
| Physiotherapy | Yes | Yes | € 38.89 | Per visit |
| Behavioral therapy (proxy price for mental health basic caregiver) | Yes | Yes | € 121 | Per visit |
| General practitioner | Yes | Yes | € 43.31 | Per visit |
| Medical service (proxy price of GP) | Yes | Yes | € 43.31 | Per visit |
| Occupational therapy | Yes | Yes | € 24.32 | Per visit |
| Speech therapy | Yes | Yes | € 40.93 | Per visit |

| **Product** | **Applicable for type of continence care** | | **Price (2022) € incl. taxes** | **Unit** |
| --- | --- | --- | --- | --- |
|  | **SCC** | **RCC** |  |  |
|  |  |  |  |  |
| Other therapists | Yes | Yes | Various prices, see below | Per visit |
| Outpatient clinic visit | Yes | Yes | € 120 | Per visit |
| Hospital day treatment | Yes | Yes | € 335 | Per visit |
|  |  |  |  |  |
| **Other healthcare costs** | | | | |
| Days in hospital | Yes | Yes | € 644 | Per night |
| Ambulance | Yes | Yes | € 528 | Per ride |
| Emergency room | Yes | Yes | € 258 | Per visit |
| ***Type of other care as indicated in open questions*** | | | | |
| Dental care | Yes | Yes | € 88.42 | Per visit |
| Disability doctor | Yes | Yes | € 92.21 | Per visit |
| Bartimeus, visual care | Yes | Yes | € 120 | Per visit |
| Dietician | Yes | Yes | € 24,70 | Per visit |
| Pedicure | Yes | Yes | € 50 | Per visit |
| Splint specialist | Yes | Yes | € 120 | Per visit |
| Wound care | Yes | Yes | € 43.31 | Per visit |
| Unclear type of care, outpatient clinic as proxy | Yes | Yes | € 120 | Per visit |
| Art therapist (proxy price for mental health basic caregiver) | Yes | Yes | € 121 | Per visit |
| **Cost for participant and family** | | | | |
| Travel cost to general practitioner, medical service, physiotherapist, occupational therapist, speech therapist, and behavioral therapist | Yes | Yes | € 0 | Per visit |
| Travel costs to the outpatient clinic | Yes | Yes | € 30.43 | Per visit |
| Travel costs to the hospital | Yes | Yes | € 30.43 | Per visit |
| Travel costs to the emergency room | Yes | Yes | € 30.43 | Per visit |
| Travel costs to dental care | Yes | Yes | € 17.59 | Per visit |
| Travel costs to disability doctor | Yes | Yes | € 30.43 | Per visit |
| Travel costs to Bartimeus, visual care (Zeist) | Yes | Yes | € 476.02 | Per visit |
| Travel costs to a dietician | Yes | Yes | € 17.59 | Per visit |
| Travel costs to a pedicure | Yes | Yes | € 17.59 | Per visit |
| Travel costs to the splint specialist | Yes | Yes | € 30.43 | Per visit |

| **Product** | **Applicable for type of continence care** | | **Price (2022) € incl. taxes** | **Unit** |
| --- | --- | --- | --- | --- |
|  | **SCC** | **RCC** |  |  |
| Travel costs to wound care | Yes | Yes | € 30.43 | Per visit |
| Unclear type of care, outpatient clinic as proxy | Yes | Yes | € 30.43 | Per visit |
| Travel costs to an art therapist | Yes | Yes | € 30.43 | Per visit |
| Other participants and family costs | Yes | Yes | € 0 | Per day |

Intervention Costs

Staff Costs for Continence Care

With the continence diaries, data were collected on caregivers' time spent on continence care and the number of weekly incontinence material changes. If a second colleague was helping, this time was added. Each participant's weekly time spent on continence care could be calculated during each measurement.

The value of the time spent was based on the gross salary of a function of an ‘average employee’ providing this continence care. A survey was conducted among the professional caregivers involved in continence care for the target population. The functions varied from ‘Begeleider 1’ to ‘verpleegkundige 3’. Although ‘Begeleider 1’ is the largest group, we argued that using their average wage as a representation of the costs was an underrepresentation. The second largest group was ‘Begeleider 3’, and it was also the ‘median’ group. For corroboration, we also calculated the weighted average wage of all functions; this average is very similar to the average wage of ‘Begeleider 3’. Therefore, the average wage of ‘Begeleider 3’ was used to calculate the staff costs for providing continence care, as per the Dutch guideline for EEH.

Begeleider 3 belongs to ‘functiegroep 35’, resulting in a gross salary of €2,659 per month (in 2022 EUR, based on the median plus 1 standard deviation of the salary range). The full-time work week is 36 hours, similar to professional caregivers in hospital settings. Therefore, the number of working hours for hospital professional caregivers was used as a proxy for professional caregivers in disability care, which is 1,543 hours.

As continence care must be provided 24/7, we must consider the irregularity allowance equal to 26%, based on a week without holidays (Table 2).

Table S2: Irregularity allowance in disability care

| **Hours** | **Total hours** | **Percentage irregularity allowance** |
| --- | --- | --- |
| **Monday – Friday** |  |  |
| Between 0-6h | 6 h, 5 days = 30 hours | 44% |
| Between 6-7h | 1 h, 5 days = 5 hours | 22% |
| Between 7-20h | 13 h, 5 days = 65 hours | 0% |
| Between 20-22h | 2 h, 5 days = 10 hours | 22% |
| Between 22h and 0h | 2 h, 5 days = 10 hours | 44 % |

| **Hours** | **Total hours** | **Percentage irregularity allowance** |
| --- | --- | --- |
| **Saturday** |  |  |
| Between 0-6h | 6 h, 1 day = 6 hours | 49% |
| Between 6-8h | 2 h, 1 day = 2 hours | 38% |
| Between 8-12h | 4h, 1 day = 4 hours | 0% |
| Between 12-22h | 10h, 1 day = 10 hours | 38% |
| Between 22h and 0h | 2 h, 1 day = 2 hours | 49% |
| **Sundays and bank holidays** |  |  |
| 0-24h | 24h, 1 day = 24 hours | 60% |

Additional costs, such as holidays and social security costs, were also considered. The Dutch guideline for EEH suggested that the gross salary, including the irregularity allowance, should be increased by 41% [1].

This resulted in the following personnel costs: €2,659 * 1.26 * 1.41 = €4,724 per month, multiplied by 12, equals €56,688 per year. With 1,543 workable hours per year, the cost per hour was €36.74**.**

Incontinence Material

Different brands (Abena, Absorbin, Tena) of incontinence materials were used in regular care. For each brand, the price per piece was determined based on financial data from a care organization or information from the supplier itself. To determine the average price, the prices of the most commonly used products in this study were used, which included Absorin Ultra Medium Grijs, Tena Slip Ultima Medium, and Abena M4. The prices of these products varied between €0.62 and €0.88, with an average of €0.74. This average price for regular incontinence material was used in the base case analyses.

Incontinence material with integrated sensors was used for smart continence care. The 2022 price of the most popular product, Abena Nova M4, was €1.30, including taxes. Discount prices were applied when the organization was implementing Abena Nova throughout the organization, letting the price per piece to drop to €1.08 (incl taxes), independent of the size and absorptive capacity.

Table S3: Average price of incontinence material

| **Price of incontinence material** | **Average price € (2022), including taxes** |
| --- | --- |
| Regular incontinence material | 0.74 |
| Smart continence material | 1.30 |

Disposables

Besides incontinence material, other disposables were used, such as bathing gloves, medical gloves, and wipes. Based on our field observations, the following materials were used for each incontinence material change: 1 pair of gloves and 4 disposable wash gloves. This is in line with the observations by Bliss et al. [2] studying incontinence-associated dermatitis care, who observed a median of 1 pair of medical gloves, 2 disposable wipes, and 2 wash cloths.

Table S4: Price determination of the disposables, based on the care organization’s financial data (2022)

| **Package** | **Price € (2022) including taxes** | **Price € (2022) per piece** | **Number of products used** | **Price € (2022) per incontinence material change** |
| --- | --- | --- | --- | --- |
| 200 pieces of medical gloves | 22.07 | 0.11 | 2 | 0.22 |
| 320 8-pack disposable wash gloves | 98.13 | 0.04 | 4 | 0.15 |
| **Total costs of disposables per incontinence material change** | | | | **0.37** |

The total costs of the disposables used per incontinence material change are estimated at €0.37.

Skin and Wound Care

Although overall medication costs were omitted from this study due to time constraints, we did collect data on the amount of skin and wound care provided. Costs associated with this specific ‘medication’ use were included in this study.

Based on the financial data (year 2022) of one organization, together with field observations, 4 products were identified for skin and wound care during continence care. The amounts of product used are derived from an American study [3]; for barrier film products, this is 1,1 grams per application, and for ointments, this is 6,1 grams per application.

Table S5: Cost associated with skin and wound care

| **Product (in Dutch)** | **Volume** | **Price € (2022) including taxes** | **Resource used (Zehrer 2004)** | **Price € (2022) per application** |
| --- | --- | --- | --- | --- |
| Cavilon Spray, Alcohol Vrije Barriere Film, 3M | 28 ml (21.84 gram) | € 11.45 | 1.1 gram | €0.58 |
| Sudocreme, Huidbeschermende creme, Geschikt bij Luieruitslag | 125 gr | € 6.39 | 6.1 gr | €0.31 |
| Zinkzalf, Dr Swaab | 30 gr | € 5.22 | 6.1 gr | €1.06 |
| Huidbeschermingscreme, Proshield Plus | 115 gr | € 17.86 | 6.1 gr | €0.95 |
| **Average price** | | | | **€ 0.73** |

The average price of providing wound or skincare was estimated at €0.73.

Laundry Costs

Leakages can result in a bed that needs to be changed (either a full or half bed) or clothes that need to be replaced. Additional time used for these activities was registered with the time spent on continence care; caregivers were asked to include this additional time when noting down the time. Yet, doing additional laundry because of leakages comes with additional costs, such as the costs for the detergent, electricity, water, and use of the machine.

The Dutch National Institute for Budgeting (Nibud) calculated the costs per laundry using a washing machine and a tumble dryer [4]. We assumed that one bed that fully needs to be changed is equivalent to one laundry unit, half a bed is 0.5 laundry units, and one set of clothes is 1/3 of a laundry unit. Having a consumer washing machine and tumble dryer at the location, the cost of one laundry unit was estimated at €2.58 (indexed to 2022).

Table S6: Laundry costs

| **Type of laundry** | **Price € (2022)** |
| --- | --- |
| Full bed (1 unit) | 2.58 |
| Half bed (0.5 unit) | 1.29 |
| Set of clothes (1/3 unit) | 0.86 |

Additional Cost For Smart Continence Care

For the smart continence care system to function, one needs hardware, such as clips, relays, and caregiver phones. An installation fee could also be applied. According to the quote from the supplier (2022) these costs are:

- € 36,30 (incl 21% taxes) for a clip, a participant uses one clip. A clip has an expected lifespan of 9 months
- € 60,5 (incl 21% taxes) for using relays (if returned after the 12-week research period)
- € 60,5 (incl 21% taxes) for using caregiver phones (applicable if the care organization does not have (enough) own mobile phones available, if returned after the 12-week research period, excluded for this analysis)
- € 121 (incl 21% taxes) installation fee per participant

A top-down micro-costing is used to assign these intervention costs to a single participant per day, based on the actual invoices received by the care organizations. This method was chosen because the supplier offers the possibility to negotiate pricing, for example, based on the number of locations and participants, as well as the duration of the procurement contract. Using the actual invoices ensures the most realistic estimates of the daily costs per participant.

Based on the amounts of the actual invoices, we calculated an average price per participant per day for the analyses (Table 7).

Table S7: Additional cost for smart continence care

| **Product** | **Price € (2022) per participant per day, incl taxes** |
| --- | --- |
| License fee | 6.05 |
| Relay | 0.56 |
| Clip | 0.22 |

Other Healthcare Costs

Other costs related to healthcare consist of healthcare resources and services people with profound intellectual and multiple disabilities use, not directly related to continence care. Most items had a reference price as stated in the Dutch guideline for EEH [1]. Yet, for one item, costs had to be calculated to avoid double-counting. These are the costs associated with ‘inpatient stay in disability care, excluding continence care’.

Stay Disability Care, Excluding Continence Care

The participants in this study reside in disability care facilities, and most also attend a day activity center or receive services at their residence. The Dutch guideline for EEH [1] gives various reference prices for different target groups in disability care. These costs also include the cost of continence care. Therefore, we had to perform our own cost price research related to the continence care part.

For the inpatient stay, the average reference price, including daycare, is: € 349. This includes the costs for continence care, which is estimated at €28.89 per day (see Table 8). The inpatient stay price, excluding continence care, is, therefore, €319.78. This is based on deducting the average price of providing continence care, as measured in this study for 6 care organizations at T0.

Table S8: Cost of continence care on baseline, based on T0 in both groups

|  |  | **Average number of incontinence material changes per week** | **Price € (2022)** | **Costs per week € (2022)** |
| --- | --- | --- | --- | --- |
| Staff used for continence care moment | 13.43 minutes | 21.044 | 36.74 | € 173.07 |
| Incontinence material costs |  | 21.044 | 0.74 | € 15.573 |
| Disposable costs |  | 21.044 | 0.33 | € 6.94 |
| Full bed laundry | 0.057 | 21.044 | € 2.58 | € 3.115 |
| Half-bed laundry | 0.074 | 21.044 | € 1.29 | € 1.996 |
| Clothes laundry | 0.084 | 21.044 | € 0.86 | € 1.511 |
| **Total cost of continence care per week:** | | | | € 202.21 |
| **Continence care cost per day:** | | | | € 28.89 |
| **Average day cost of disability care, excluding continence care, including activity center** | | | | **€ 319.78** |

Other Healthcare Cost Items

The Medical Cost Consumption Questionnaire (iMTA MCQ) is used to gather information on resource use and other health-related costs. The questionnaire is adjusted to make it relevant for our target group. such as including ‘medical service’ and ‘behavioral therapy’, and excluding costs as ‘inpatient cost in elderly care or a mental care institution. For most types of care, the reference prices are available.

Table S9: Reference prices for health care services

| **Type of care** | **Price € (2022)** |
| --- | --- |
| General practitioner | 43.31 |
| Medical service (proxy price of GP) | 43.31 |
| Physiotherapy | 38.89 |
| Occupational therapy | 24.32 |
| Speech therapy | 40.93 |
| Behavioral therapy (proxy price for mental health basic caregiver, table 4-15-4) | 121 |
| Emergency room | 258 |
| Ambulance | 528 |
| Outpatient clinic visit | 120 |
| Hospital day treatment | 335 |
| Hospital night | 644 |

The open-ended questions in the questionnaire allowed for responses from other types of healthcare providers. The prices for these answers are based on the NZA (Dutch Healthcare Authority) tariffs, prices mentioned by health insurance, or proxy prices used (table 10). For each participant, individual answers were reviewed to avoid double-counting.

Table S10: Prices of those not available in the Dutch Guideline, thus having our own price research

| **Type of care. open answer** | **Source to determine the price** | **Assumption** | **Price € (2022)** |
| --- | --- | --- | --- |
| Dental care. ‘Tandarts, preventive assistente, mondhygienist’ | Prestatie- en tariefbeschikking tandheelkundige zorg - TB/REG-22610-02 - Nederlandse Zorgautoriteit (overheid.nl) | Periodic consult  Additional medical questions  15 min cleaning tooth  *Based on a basic visit as a reference price* | 23.45  23.45  3*13.84  88.42 |
| Doctor specialized in intellectual disabled (AVG arts) | Beleidsregel prestatiebeschrijvingen en tarieven modulaire zorg 2024 - BR/REG-24122a - Nederlandse Zorgautoriteit (overheid.nl) | Max tariff 1 hour of treatment of AVG doctor. €193.03 (2024 prices).  Assumption; one visit is 30 minutes. | 184.41 per hour (2022 prices)  92.21 per visit |
| Bartimeus. institute specialises in care for the visually disabled. | Verdiepingsmodule 2024 | Proxy price, regular outpatient clinic visit | 120 |
| Dietician | Verdiepingsmodule 2024 |  | 24.70 |
| Pedicure | Vergoeding van medische pedicure - Independer | Assumed the higher end of the price range mentioned | 50 |
| Splint specialist (Spalkman) | Verdiepingsmodule 2024 | Proxy price, regular outpatient clinic visit | 120 |
| Woundcare | Verdiepingsmodule 2024 | Proxy price. regular GP visit | 43.31 |
| Care, which was not possible to define the actual type | Answers ‘PPM’. | Assumed proxy of outpatient clinic visit | 120 |
| Art therapist | Verdiepingsmodule 2024 | proxy price for mental health basic caregiver | 121 |

Other Participant and Family Costs

Researcher VvC completed several site visits and observations within several care organizations, including residences, day activity centers, and night care teams. During regular care and when using smart continence care, informal discussions with professional caregivers taught us that there were older participants whose families did not regularly visit. In other cases, participants spent a day with family outside the facility or spent the night with them. Furthermore, participants visited day activity centers to receive day activities, while others received them at the residence (also due to COVID-19 measures). Continence care, or leakages, was not mentioned as a factor influencing the type and amount of these activities. Furthermore, it was not yet possible to use smart continence care outside the facility, as it required a receiver (with a power bank) to travel with the participant, and a family member needed access to the app receiving the notifications, which they did not have. Due to administrative and privacy concerns, this was difficult to organize. Data from the general questionnaire, collected after 12 weeks of smart continence care and regular continence care, did not reveal any changes in the day program related to the type of care provided. Due to the nature of the disability, there was no change in productivity loss or absenteeism, as these people did not have (unpaid) work. Additionally, we did not expect any changes for the family members due to a change in continence care, as the participants reside within a healthcare facility. Therefore, we argue that there were no significant changes in other costs for participants and their families, except for travel costs.

Travel Costs

In this study, the participants lived at a care organization. Within these organizations, specialists were available in the organization, thus minimizing travel costs. We state that there were no travel costs for a visit to the general practitioner, medical service, physiotherapist, occupational therapist, speech therapist, and behavioral therapist.

For a visit to another specialist, travel costs apply. Due to the nature of the disability, these patients traveled by taxi, thus incurring higher travel costs than the average. The Dutch guideline for EEH states that taxi costs are €3.36 as a start cost and €2.47 per kilometer. For the travelled distance, we used the average distances as stated in the Dutch guideline for EEH.

This results in the following costs:

*Table S11: Travel costs*

| **Visit to** | **Average distance (km)** | **Breakdown of cost** | **Price (2022) €** |
| --- | --- | --- | --- |
| Outpatient clinic | 4.8 | 2*(3.36 + (2.47 * 4.8)) | € 30.43 |
| Hospital | 4.8 | 2*(3.36 + (2.47 * 4.8)) | € 30.43 |
| Emergency room | 4.8 | 2*(3.36 + (2.47 * 4.8)) | € 30.43 |
| *Other types of care* | | | |
| Dental care | 2.2 (proxy is physiotherapy practice) | 2*(3.36 + (2.47 * 2.2)) | € 17.59 |
| Disability doctor | 4.8 (proxy is hospital) | 2*(3.36 + (2.47 * 4.8)) | € 30.43 |
| Bartimeus. visual care (Zeist) | 95 KM one way trip | 2*(3.36 + (2.47 * 95)) | € 476.02 |
| Dietician | 2.2 (proxy is physiotherapy practice) | 2*(3.36 + (2.47 * 2.2)) | € 17.59 |
| Pedicure | 2.2 (proxy is physiotherapy practice) | 2*(3.36 + (2.47 * 2.2)) | € 17.59 |
| Splint specialist | 4.8 (proxy is hospital) | 2*(3.36 + (2.47 * 4.8)) | € 30.43 |
| Wound care | 4.8 (proxy is hospital) | 2*(3.36 + (2.47 * 4.8)) | € 30.43 |
| Art therapist | 4.8 (proxy is hospital) | 2*(3.36 + (2.47 * 4.8)) | € 30.43 |
| Unclear type of care, out patient clinic as proxy | 4.8 (proxy is hospital) | 2*(3.36 + (2.47 * 4.8)) | € 30.43 |

References

1. Zorginstituut Nederland. Guideline for economic evaluations in healthcare 2024
2. Bliss DZ, Zehrer C, Savik K, Smith G, Hedblom E. An economic evaluation of four skin damage prevention regimens in nursing home residents with incontinence: economics of skin damage prevention. J Wound Ostomy Continence Nurs. 2007 Mar-Apr;34(2):143-52; discussion 52. PMID: 17413828. doi: 10.1097/01.Won.0000264825.03485.40.
3. Zehrer CL, Lutz JB, Hedblom EC, Ding L. A comparison of cost and efficacy of three incontinence skin barrier products. Ostomy Wound Manage. 2004 Dec;50(12):51-8. PMID: 15632456.
4. Nibud. Richtlijn waskosten zorginstellingen [Guideline laundry costs care organizations] 2012.
