# Supplementary material for: Smart Continence Care for People With Profound Intellectual and Multiple Disabilities Within Dutch Residential Care Facilities: Economic Evaluation Alongside a Cluster Randomized Trial
Source: J Med Internet Res. 2025 Oct 10;27:e72017. doi: 10.2196/72017 (PMC12552815; doi:10.2196/72017)
Supplement: Multimedia Appendix 4 [file jmir_v27i1e72017_app4.docx]

The primary (base-case) analyses are performed according to the intention-to-treat principle, using linear mixed regression methods via R software and the packages BCEA, nlme and lme4 (R version 4.0+; R Foundation for Statistical Computing, Vienna, Austria) for both effectiveness and cost measures, computed over T0-T2. The model accounted for the clustering of the data (at the location level) while also controlling for potential imbalances between arms in some baseline variables. The general specification of the model fitted to the two clinical effectiveness outcomes of interest for the economic evaluation (number of leakages and IMC per week) is:

Outcome_𝑖𝑗𝑙_=α_0_+α_1_×T1+α_2_×T2+α_3_×T1×arm+α_4_×T2×arm+𝜈_𝑗_+𝜂_𝑙_+𝜀_𝑖𝑗_,

where Outcome_𝑖𝑗𝑙_ denotes the effectiveness outcome variable measurement collected in location 𝑙 at time 𝑗 for individual 𝑖*,* while 𝜈*_j_*, *n_l_,* and 𝜀_𝑖𝑗_, respectively, denote the time-specific, location-specific random effect term and the residual error component in the model, each assumed to be normally distributed with mean 0 and constant variance. The vector of fixed-effect regression coefficients is denoted with **α *=*** $\left\{ \alpha_{0}, \ldots,\alpha_{4} \right\}$ with the following predictor variables being included in the model: the time indicators T1 and T2 (with T0 as the reference), the arm indicator (arm), and the interaction terms between these variables.

For total societal costs (TSC), category A, the model is specified as:

TSC_𝑖𝑙_=𝛽_0_+𝛽_1_×arm+𝛽_2_×n IMCT0+𝛽_3_×AVGTCT0+𝜂_𝑙_+𝜀_𝑖𝑗_,

where TC_𝑖𝑙_ denote the total cost outcome variable measurement computed over T2-T0 in location 𝑙 for individual 𝑖*,* while *n_l_,* and 𝜀_𝑖𝑗_ denote the location-specific and residual error component in the model, each assumed to be normally distributed with mean 0 and constant variance. The vector of fixed-effect regression coefficient is denoted with **β *=*** $\left\{ \beta_{0}, \ldots,\beta_{3} \right\}$, and the following predictor variables are included in the model: the arm indicator (arm), the baseline number of IMC (IMCT0) and average duration of continence care (AVGTCT0). The last two predictors are included to control for potential baseline imbalances between arms which, at least partially, reflect intervention costs (i.e. their values at T1 and T2 are used in cost calculations). Based on this formulation, the treatment effect of interest can be retrieved from the model as the estimate $\hat{\beta_{1}}$, representing the mean difference between arms (SCC-RCC) in total costs over T2-T0.

For QALYs, the model is specified as:

QALY_𝑖𝑙_=γ_0_+γ_1_×arm+γ_2_×EQ5DT0+𝜂_𝑙_+𝜀_𝑖𝑗_,

where QALY_𝑖𝑙_ denote the QALY outcome variable measurement computed over T2-T0 in location 𝑙 for individual 𝑖*,* while *n_l_,* and 𝜀_𝑖𝑗_ denote the location-specific and residual error component in the model, each assumed to be normally distributed with mean 0 and constant variance. The vector of fixed-effect regression coefficient is denoted with **γ *=*** $\left\{ \gamma_{0}, \ldots,\gamma_{2} \right\}$, and the following predictor variables are included in the model: the arm indicator (arm), the baseline EQ-5D utility scores (EQ5DT0). Based on this formulation, the treatment effect of interest can be retrieved from the model as the estimate $\hat{\gamma_{1}}$, representing the mean difference between arms (SCC-RCC) in QALYs over T2-T0.
