# Supplementary material for: Smart Continence Care for People With Profound Intellectual and Multiple Disabilities Within Dutch Residential Care Facilities: Economic Evaluation Alongside a Cluster Randomized Trial
Source: J Med Internet Res. 2025 Oct 10;27:e72017. doi: 10.2196/72017 (PMC12552815; doi:10.2196/72017)
Supplement: Multimedia Appendix 5 [file jmir_v27i1e72017_app5.docx]

Figures S1-S16 show the cost-effectiveness planes for each outcome measure analyzed: leakages, incontinence material changes (IMC) and quality-adjusted life years (QALY) for the base case, sensitivity (SA) and subgroup and scenario (SS) analyses. For the cost-utility analyses, with QALY as an outcome, the cost-effectiveness acceptability curve (CEAC) is also displayed. When a suggested value for the willingness to pay (WTP) threshold for evaluating the cost-effectiveness of the intervention with respect to a specific health outcome is not available from the literature or not recommended to be used, the CEAC is not displayed.

The sensitivity analyses varying the price of smart continence care (SCC) are based on the new pricing and service model of the supplier (dated October 2024). In the base case scenario, the old pricing model, the license fee was € 6.05 and included services such as training and installation. In the new pricing model, these services are invoiced separately. In the ‘small organization, small implementation’ scenario (SA3, SA4), the day tariff is estimated at €3.80, including the license fee, the clip costs, costs for relays, and service costs for installation, support and training. For the ‘large organization, large implementation’ scenario (SA5, SA6), the day tariff is estimated at €1.94. This organization is eligible for a larger discount as it also purchases regular continence materials for its residents not using SCC. Table S1 shows details of these scenarios.

Table S1: Price scenarios based upon new pricing model. All costs are presented in EUROS. Conversion rate of EUR €1=US $1.05469 (as of December 6, 2024).

|  | **Small organization, small implementation** | **Large organization, large implementation** |
| --- | --- | --- |
| **Situation** | | |
| Number of individuals with PIMD using SCC | 30 | 100 |
| Number of geographical locations/’residential parks (in Dutch: woonparken) | 2 | 5 |
| Number of residences (locations) | 6 | 17 |
| Number of teams involved | 6x day care teams, 2x night care team, 2x day activity center team | 17x day care teams, 5x night care team, 5x day activity center team |
| Dependency on supplier | High | Low: The care organization set up 3 regional smart diaper teams, trained to take over some of the services of the supplier |
| Duration contract, amortization period | 3 years | 3 years |
| **Hardware** **purchased** | | |
| Relays | 45 | 150 |
| Clips | 120 | 400 |
| Incontinence material | Varies per participant, based on research data, bootstrapping and imputation, same as base case analyses | |
| **Services** **purchased** | | |
| Number of License fee | 30 | 100 |
| Train the trainer programs | 0 | 3 |
| Ambassador training | 3 | 2 |
| Team training | 10 | 3 |
| Intake | 2 | 0 |
| Installation | 2 times, (for each geographical area), 3 hours each (=6 hours total) | 0 |
| Technical support year round | 6 times, 2 hours each (=12 hours total) | 0 |
| Services provided by smart diaper team within the care organization | 0 | 5x ambassador training, 24x team training, 7x intake services, and onsite support when needed, installation |
| Applicable packages, such as ‘first implementation’ and ‘training program basic’ | yes | yes |
| Applicable for discount | Yes, 5% for >20 SCC users | Yes, 10% for > 50 SCC users + 50% discount for purchasing regular incontinence materials |
| Day price services, including taxes | €3.80 | €1.94 |

Cost-Effectiveness Analyses, Clinical Outcome: Duration of Continence Care


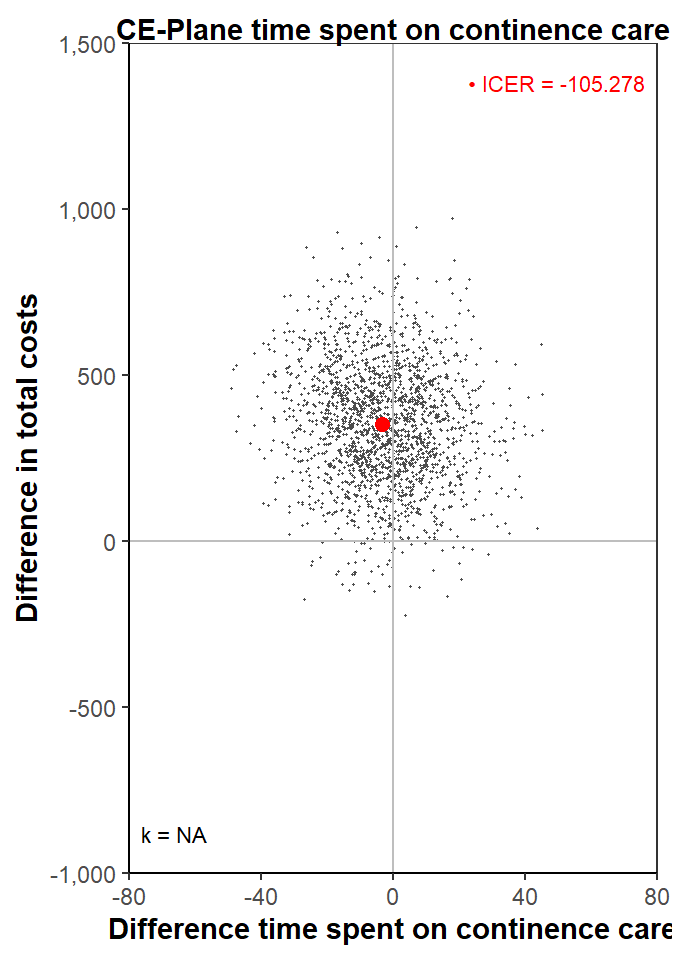


Figure S1 Clinical outcome: time spent on continence care (SA2)

Cost-Effectiveness Analyses, Clinical Outcome: Leakages

| 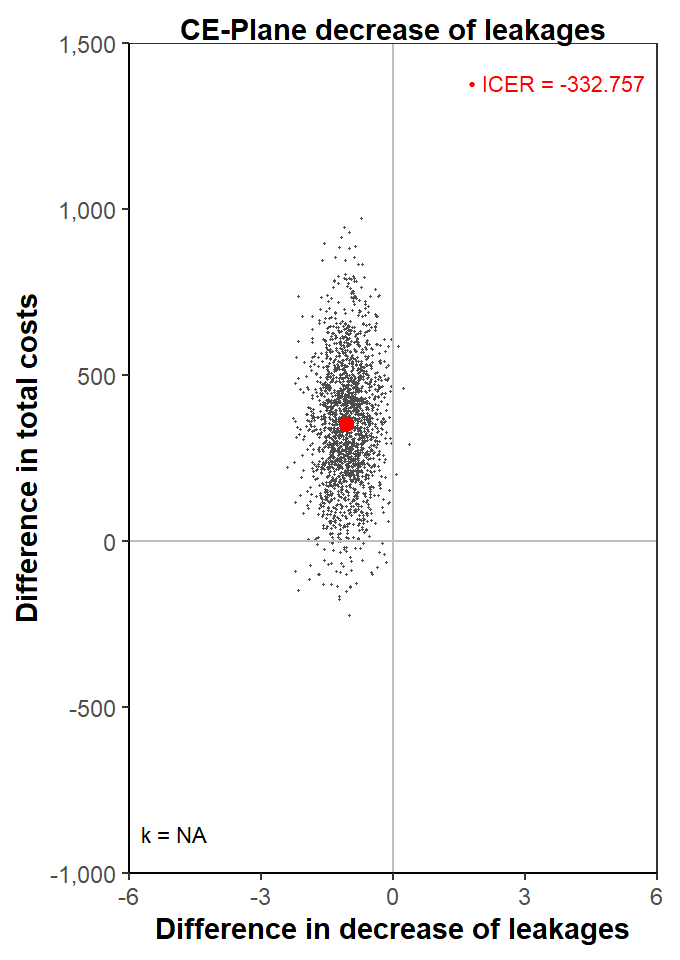  Figure S2 Base case | 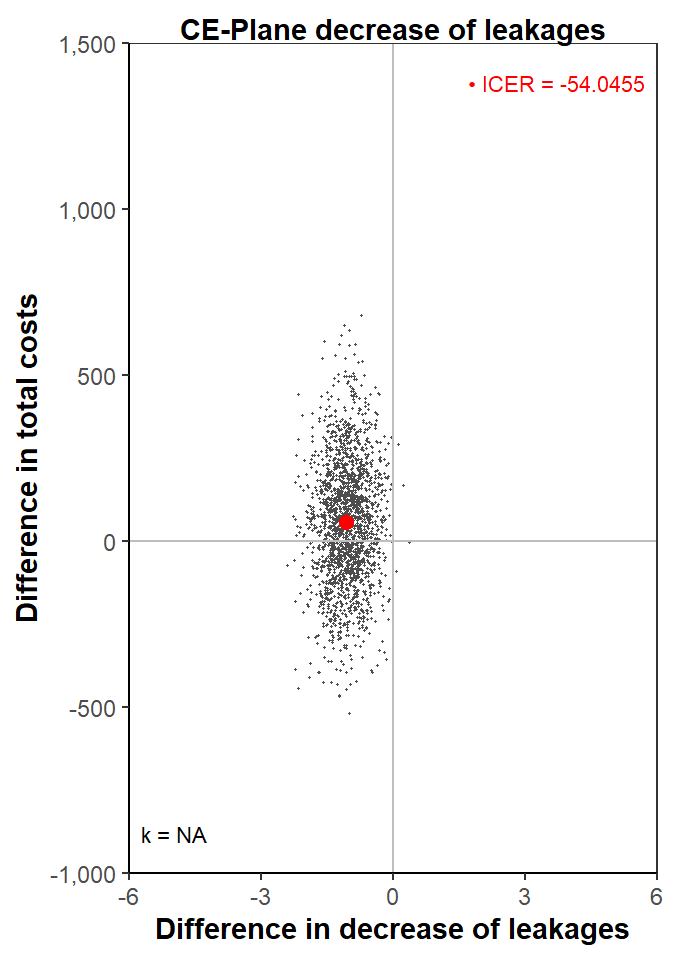 Figure S3 Sensitivity analysis, varying the price of SCC ‘small organization, small implementation’ (SA3) | 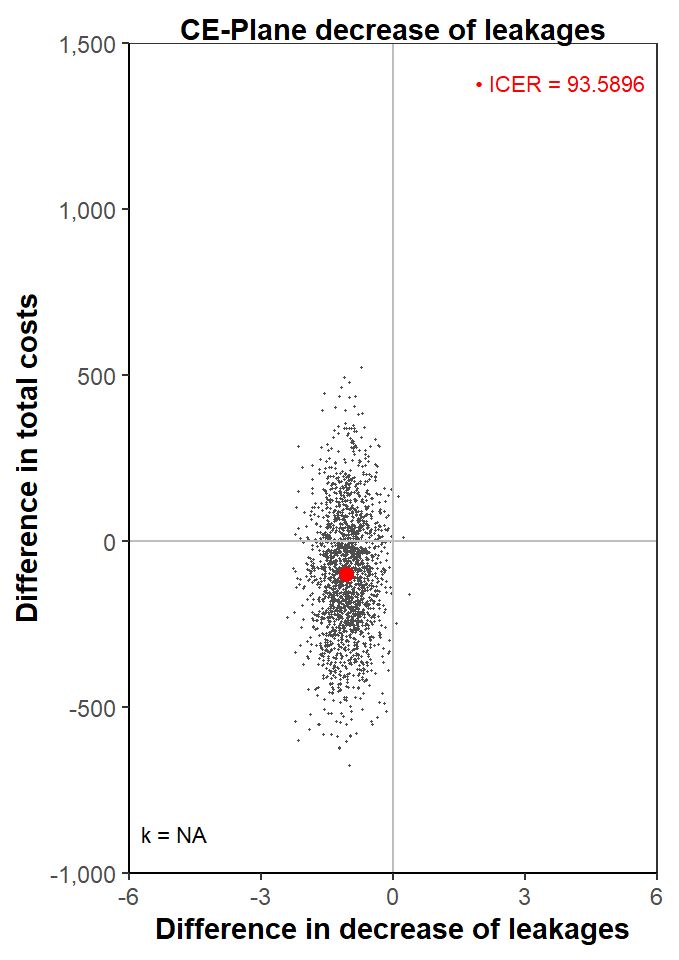  Figure S4 Sensitivity analysis, varying the price of SCC ‘large organization, large implementation’ (SA5) |
| --- | --- | --- |
| 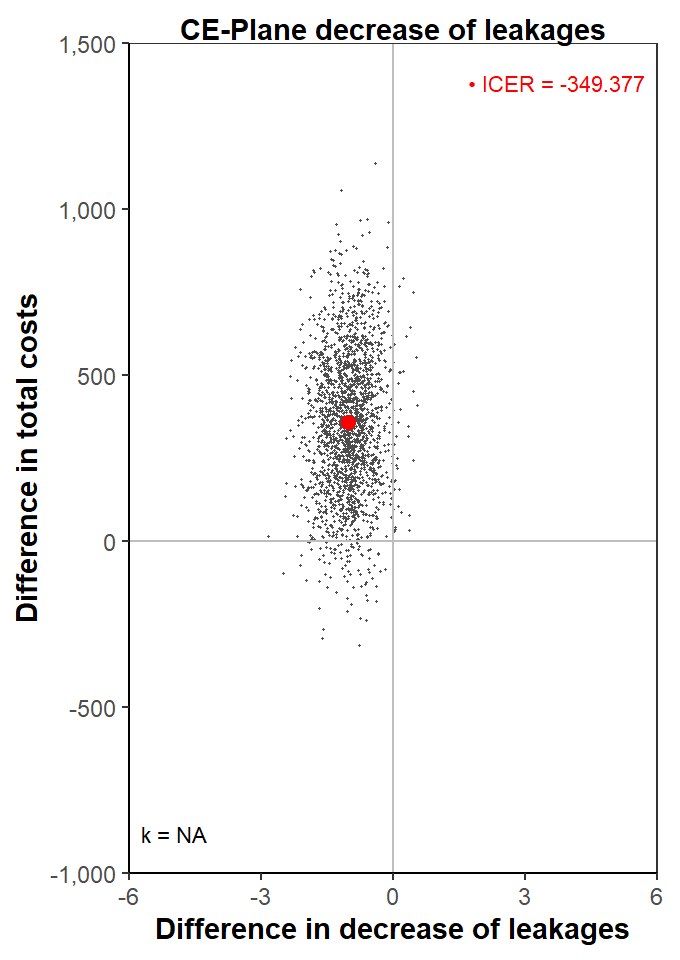  Figure S5 Per-protocol (SUA1) | 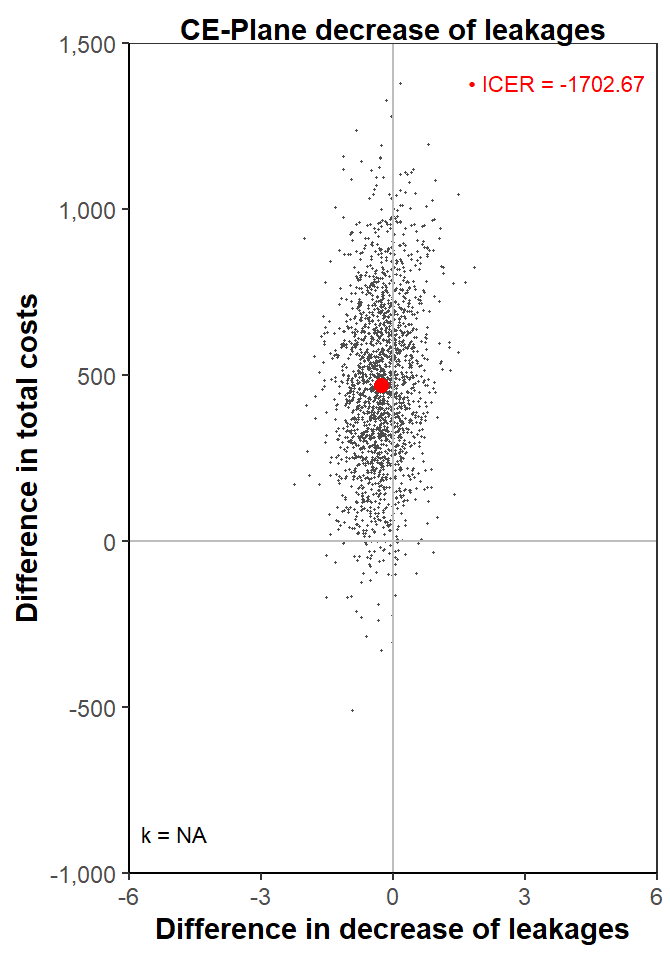  Figure S6 Excluding organization C and per-protocol (SCA1) |  |

Cost-Utility Analyses, Outcome Measure: QALY

| 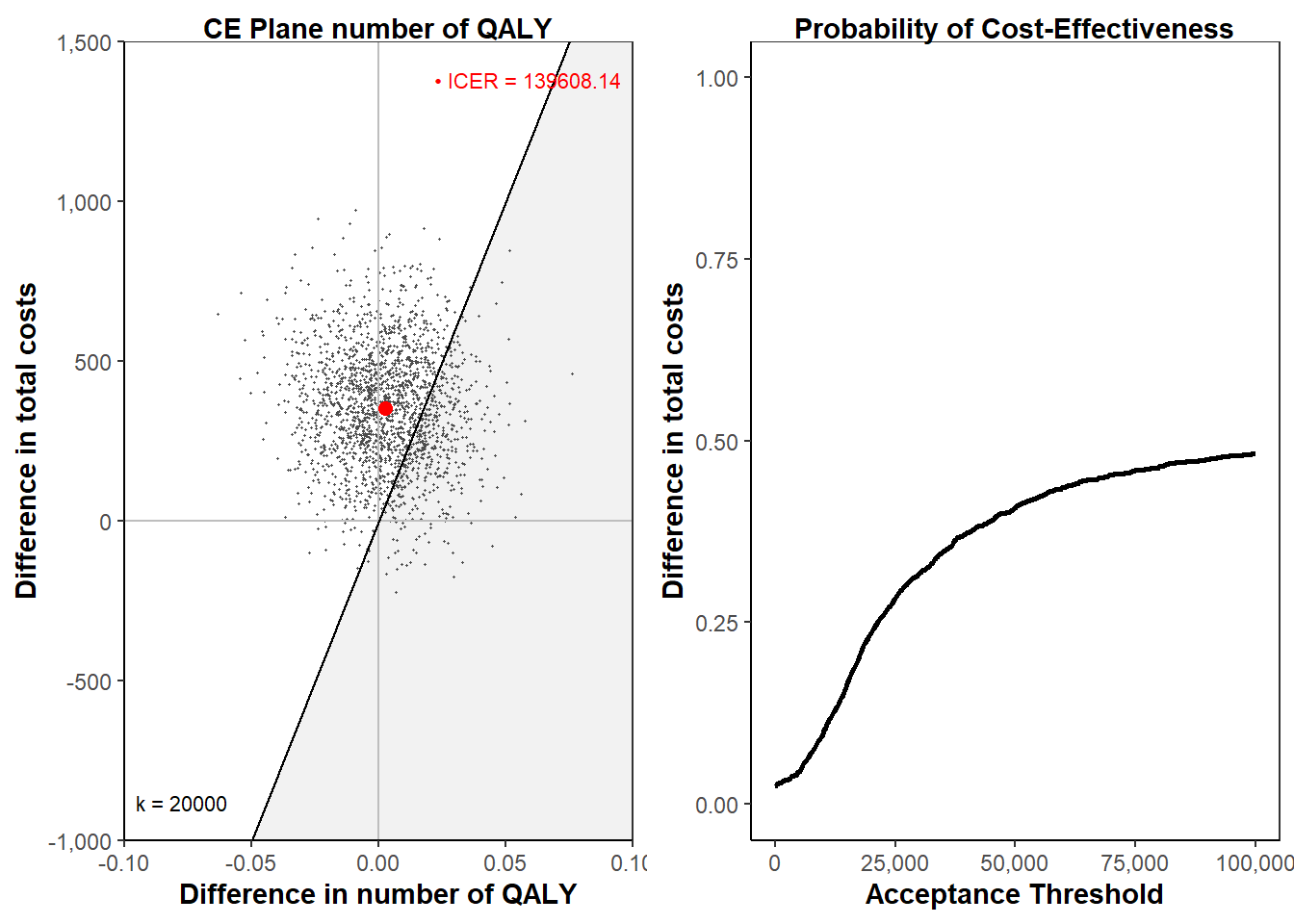  Figure S7 Base case | 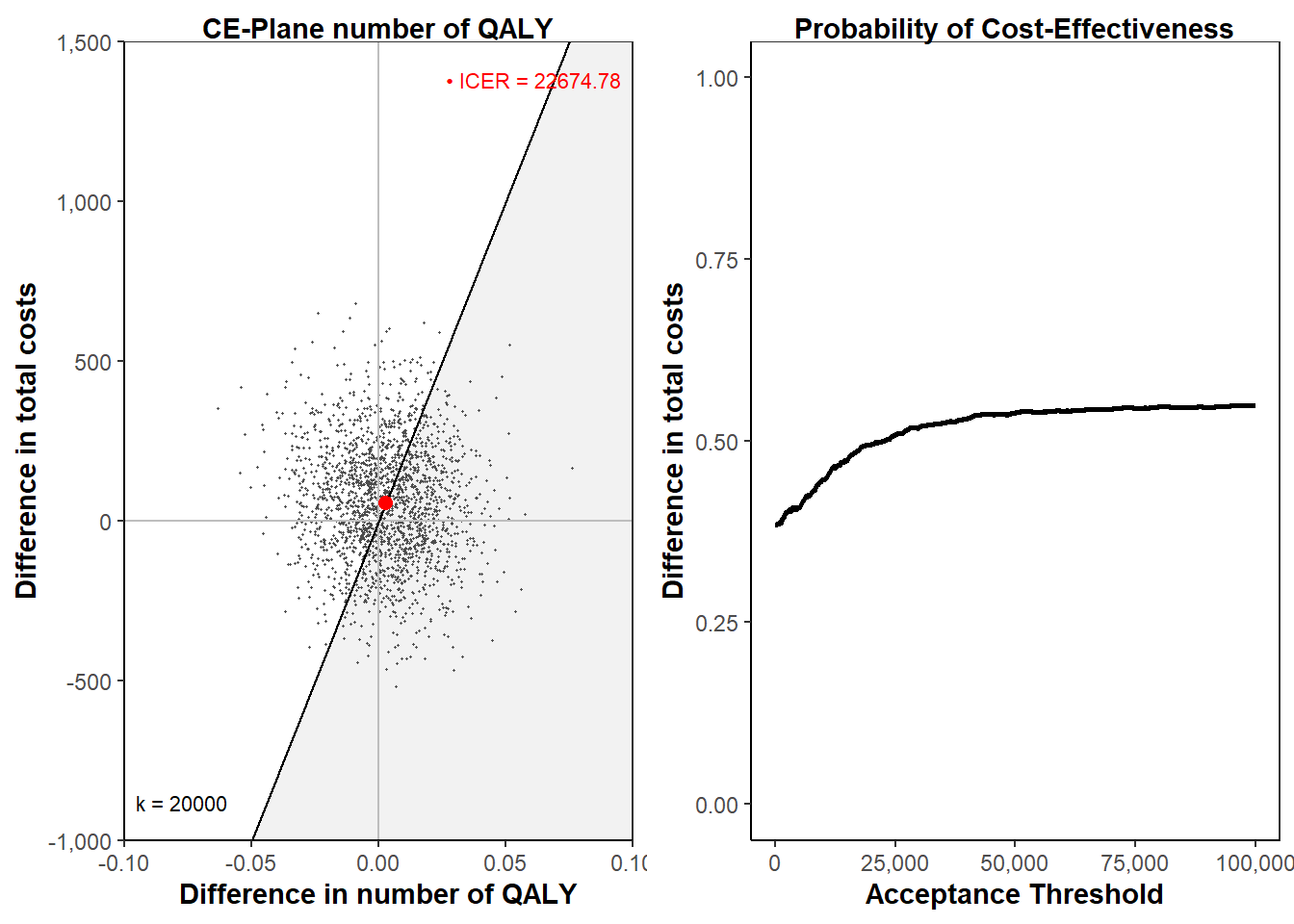  Figure S8 Sensitivity analysis, varying the price of SCC ‘small organization, small implementation’ (SA3) | 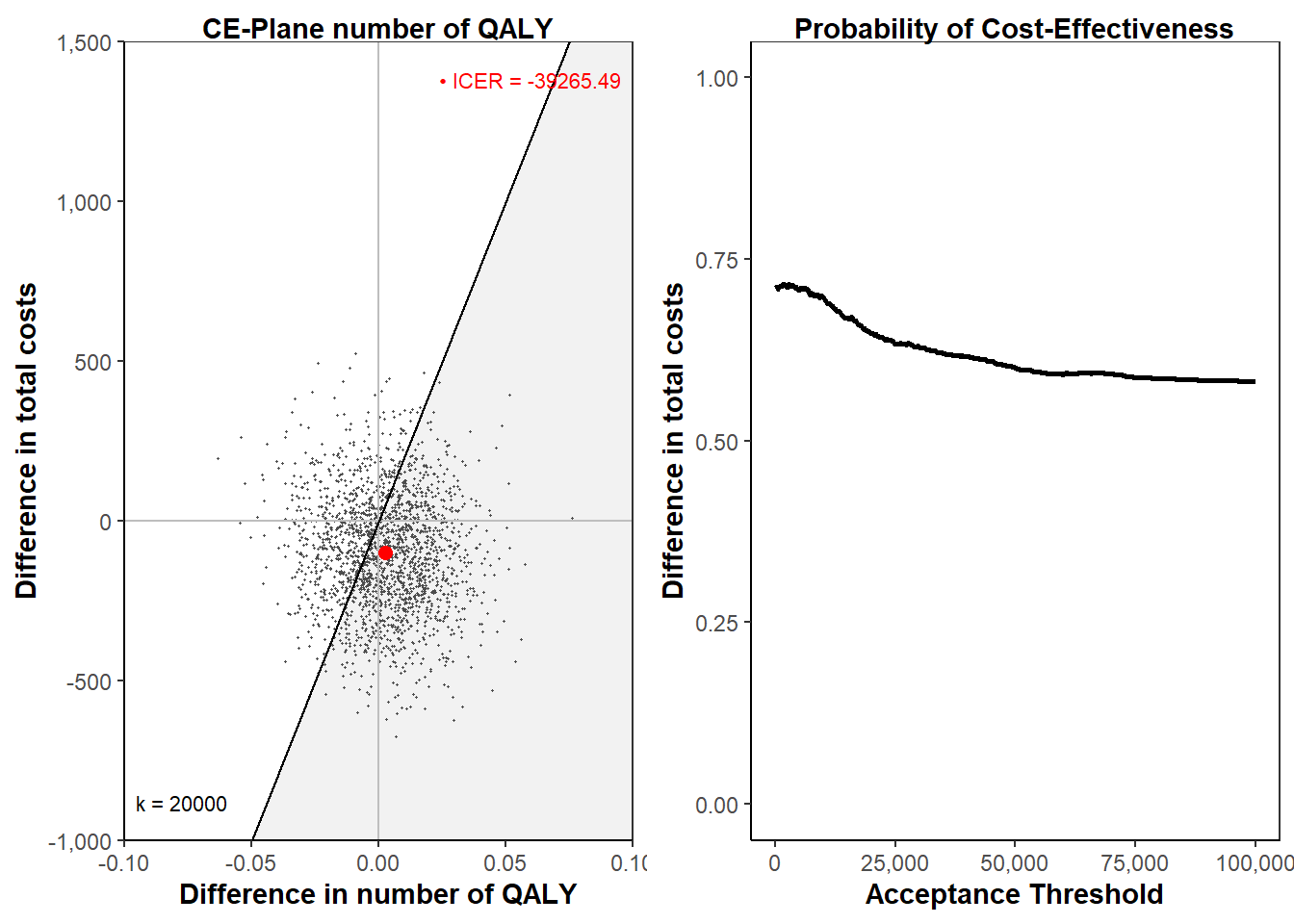  Figure S9 Sensitivity analysis, varying the price of SCC ‘large organization, large implementation’ (SA5) |
| --- | --- | --- |
| 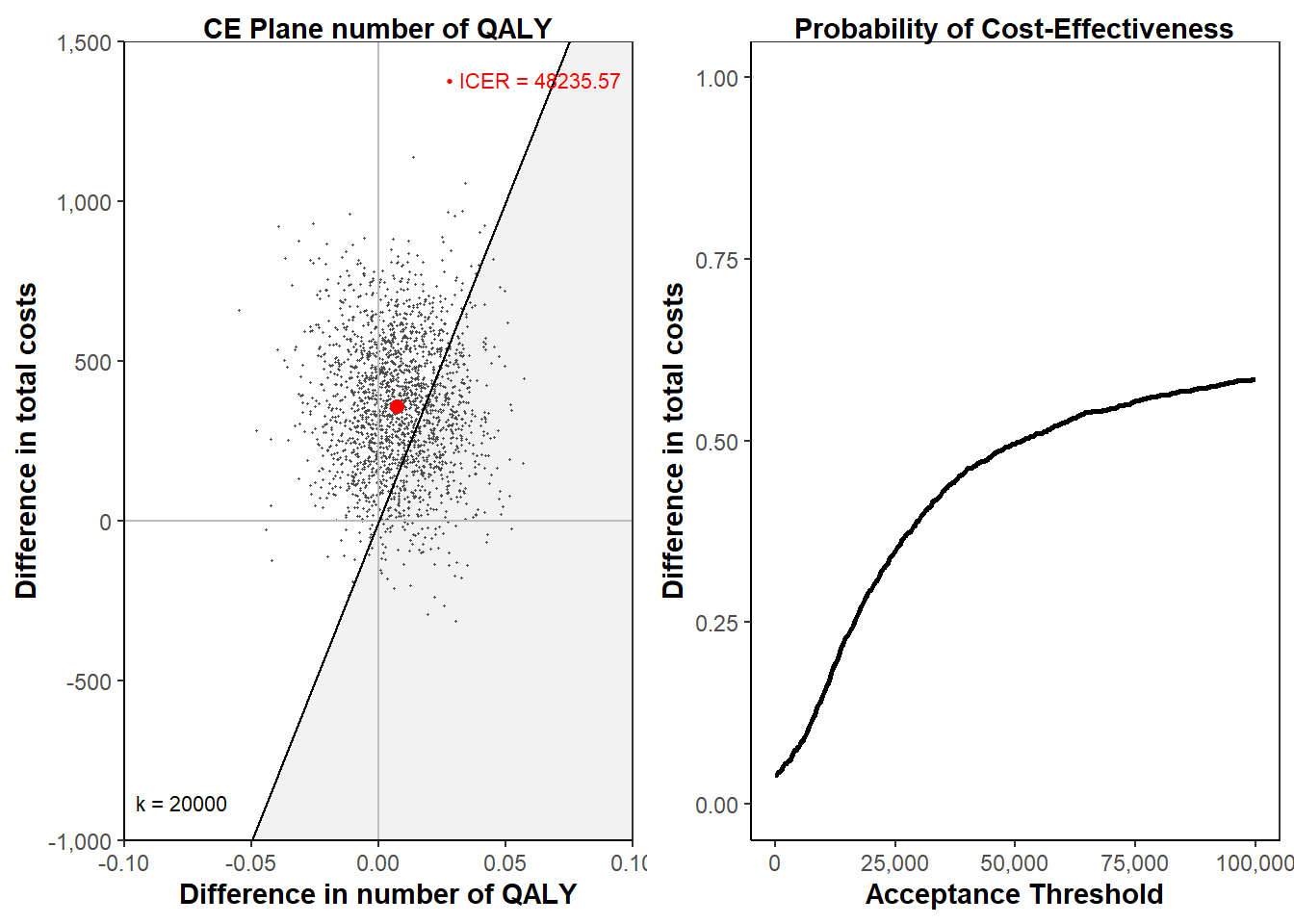  Figure S10 Per Protocol (SUA1) | 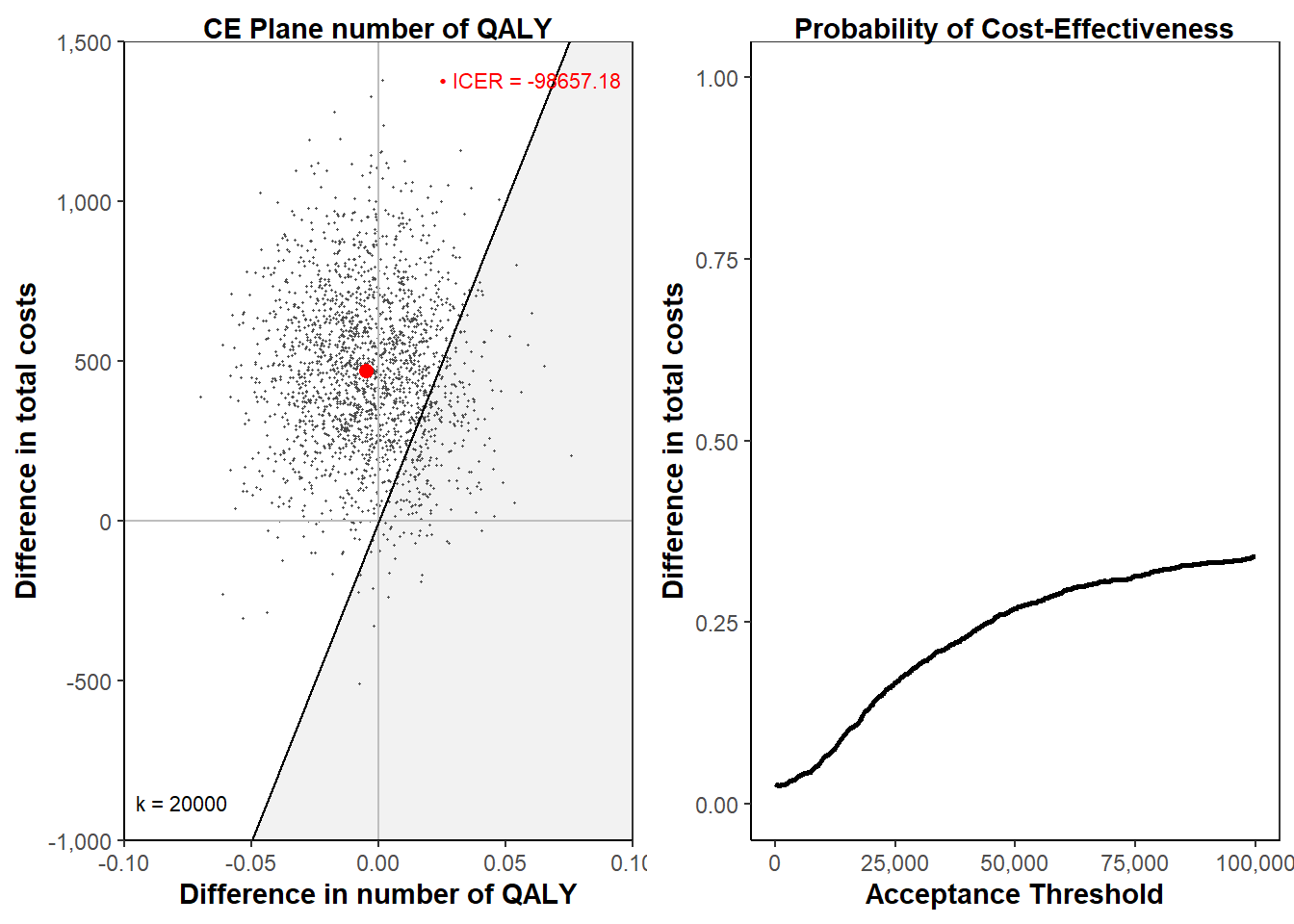  Figure S11 Excluding organization C and per-protocol (SCA1) |  |

Cost-Effectiveness Analyses, Clinical Outcome: Incontinence Material Changes

| 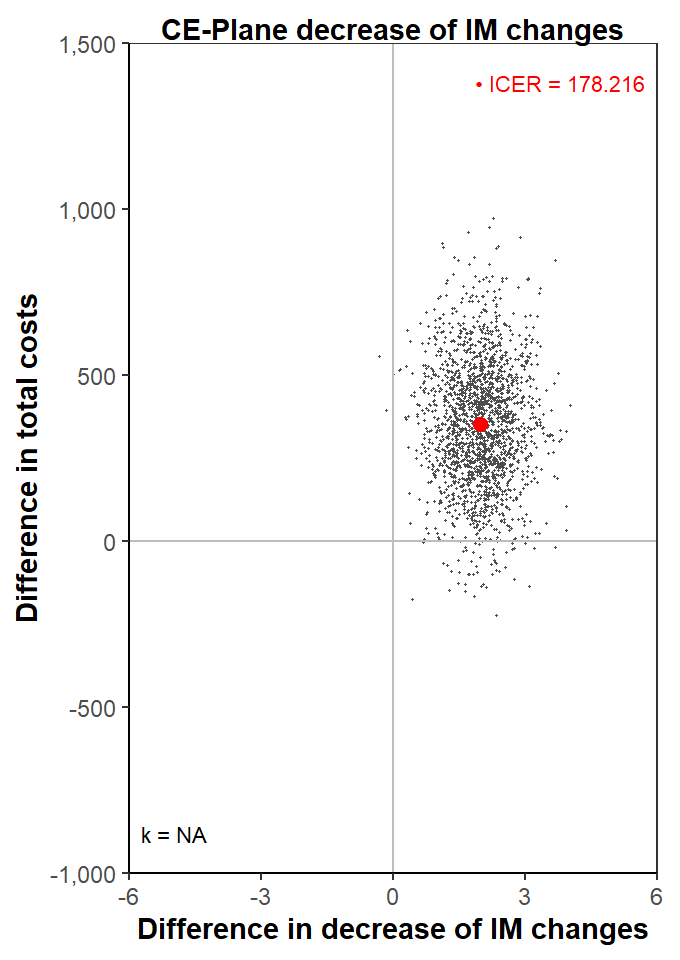  Figure S12 Different clinical outcome (SA1) | 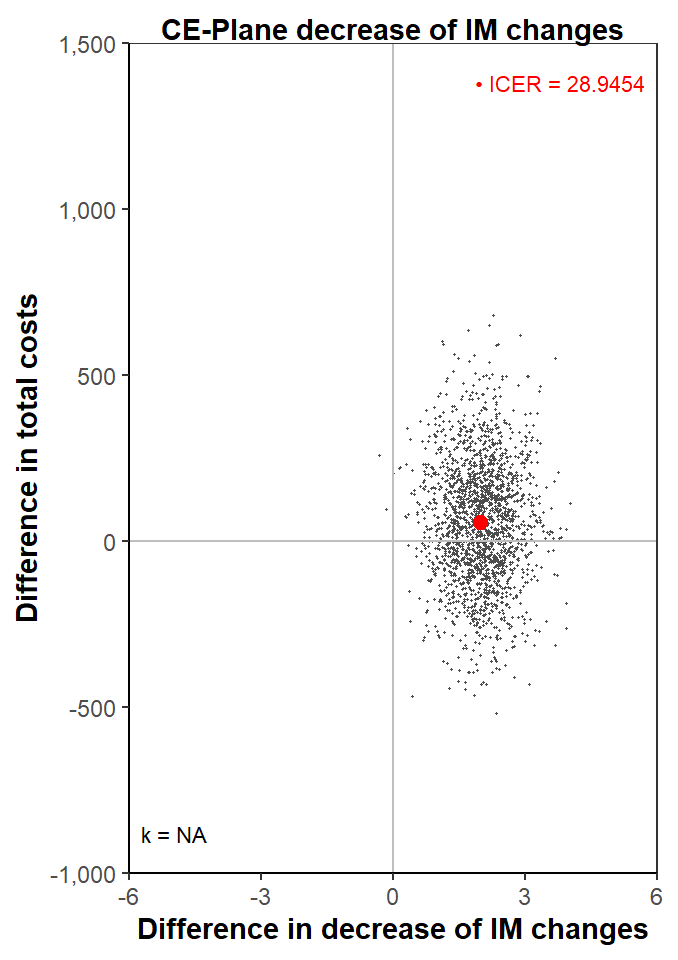  Figure S13 Sensitivity analysis, varying the price of SCC ‘small organization, small implementation’ (SA4) | 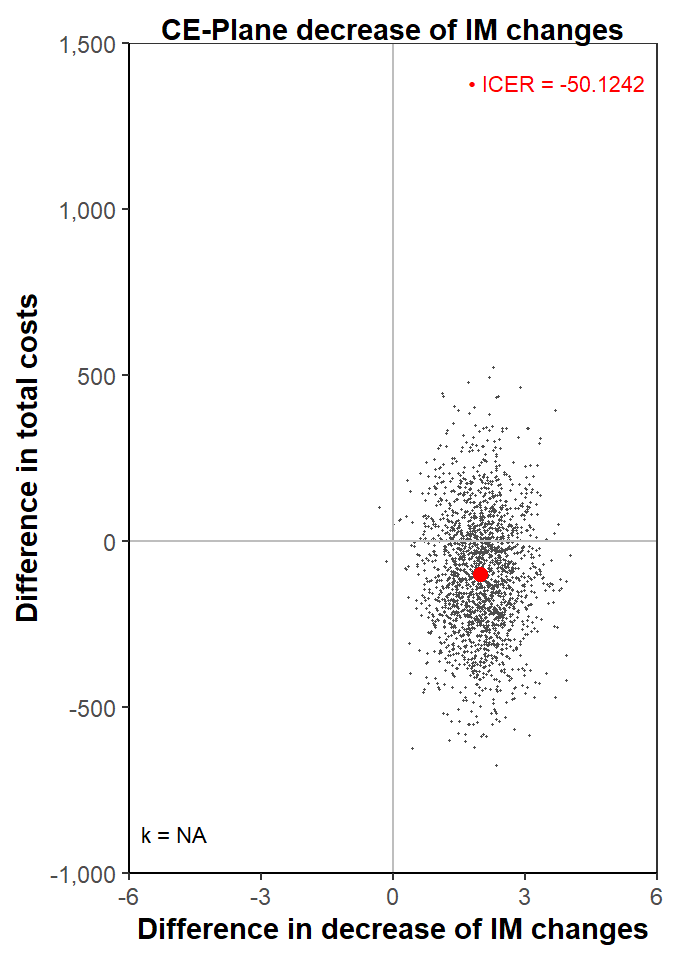  Figure S14 Sensitivity analysis, varying the price of SCC ‘large organization, large implementation’ (SA6) |
| --- | --- | --- |
| 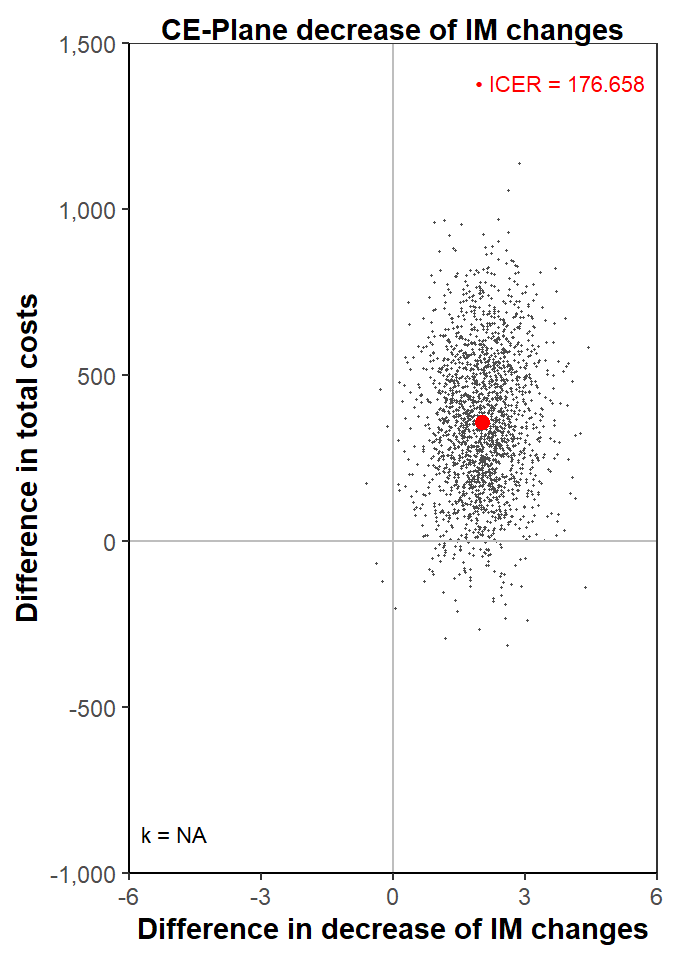  Figure S15 Per-protocol (SUA2) | 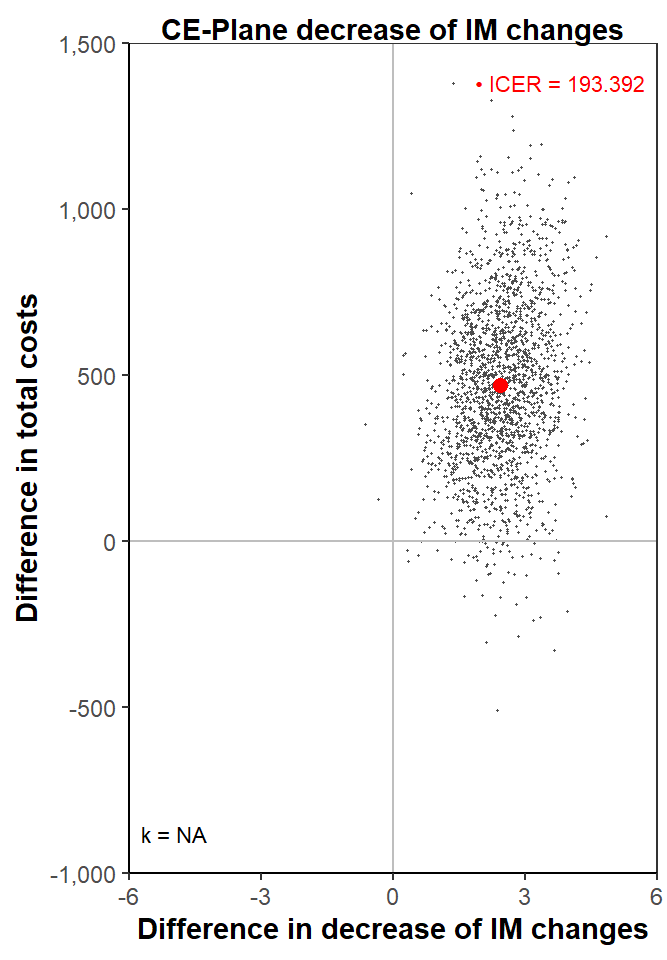  Figure S16 Excluding organization C and per-protocol (SCA2) |  |
