# Supplementary material for: Smart Continence Care for People With Profound Intellectual and Multiple Disabilities Within Dutch Residential Care Facilities: Economic Evaluation Alongside a Cluster Randomized Trial
Source: J Med Internet Res. 2025 Oct 10;27:e72017. doi: 10.2196/72017 (PMC12552815; doi:10.2196/72017)
Supplement: Multimedia Appendix 6 [file jmir_v27i1e72017_app6.docx]

Table S1: Distribution of EQ-5D-5L dimension responses at T0 and T2 for RCC and SCC group

| **Dimension** | **T0** | | | | **T2** | | | |
| --- | --- | --- | --- | --- | --- | --- | --- | --- |
|  | RCC(n=74) | | SCC(n=82) | | RCC(n=74) | | SCC(n=82) | |
|  | n | % | n | % | n | % | n | % |
| **Mobility** |  |  |  |  |  |  |  |  |
| No problems | 0 | 0 | 4 | 4.9 | 0 | 0 | 4 | 4.9 |
| Slight problem | 3 | 4.1 | 13 | 15.9 | 3 | 4.1 | 12 | 14.6 |
| Moderate problems | 3 | 4.1 | 15 | 18.3 | 2 | 2.7 | 14 | 17.1 |
| Severe problems | 7 | 9.5 | 15 | 18.3 | 8 | 10.8 | 9 | 11.0 |
| Unable to walk about | 61 | 82.4 | 33 | 40.2 | 57 | 77.0 | 32 | 39.0 |
| Missing | 0 | 0 | 2 | 2.4 | 4 | 5.4 | 11 | 13.4 |
| **Self-care** |  |  |  |  |  |  |  |  |
| No problems | 0 | 0 | 0 | 0 | 0 | 0 | 0 | 4.9 |
| Slight problem | 0 | 0 | 0 | 0 | 0 | 0 | 2 | 2.4 |
| Moderate problems | 0 | 0 | 3 | 3.7 | 1 | 1.4 | 5 | 6.1 |
| Severe problems | 2 | 2.7 | 12 | 14.6 | 5 | 6.8 | 12 | 14.6 |
| Unable to wash or dress myself | 72 | 97.3 | 66 | 80.5 | 64 | 86.5 | 52 | 63.4 |
| Missing | 0 | 0 | 1 | 1.2 | 4 | 5.4 | 11 | 13.4 |
| **Usual Activities** |  |  |  |  |  |  |  |  |
| No problems | 1 | 1.4 | 5 | 6.1 | 2 | 2.7 | 4 | 4.9 |
| Slight problem | 2 | 2.7 | 0 | 0 | 2 | 2.7 | 1 | 1.2 |
| Moderate problems | 6 | 8.1 | 10 | 12.2 | 6 | 8.1 | 8 | 9.8 |
| Severe problems | 7 | 9.5 | 26 | 31.7 | 11 | 14.9 | 15 | 18.3 |
| Unable do usual activities | 56 | 75.7 | 40 | 48.8 | 50 | 67.6 | 43 | 52.4 |
| Missing | 2 | 2.7 | 1 | 1.2 | 3 | 4.1 | 11 | 13.4 |
| **Pain / discomfort** |  |  |  |  |  |  |  |  |
| None | 14 | 18.9 | 23 | 28.0 | 15 | 20.3 | 19 | 23.2 |
| Slight | 18 | 24.3 | 26 | 31.7 | 20 | 27.0 | 18 | 22.0 |
| Moderate | 25 | 33.8 | 21 | 25.6 | 26 | 35.1 | 23 | 28.0 |
| Severe | 9 | 12.2 | 8 | 9.8 | 7 | 9.5 | 6 | 7.3 |
| Extreme | 0 | 0 | 0 | 0 | 0 | 0 | 3 | 3.7 |
| Missing | 8 | 10.8 | 4 | 4.9 | 6 | 8.1 | 13 | 15.9 |
| **Anxiety/depression** |  |  |  |  |  |  |  |  |
| None | 31 | 41.9 | 32 | 39.0 | 26 | 35.1 | 22 | 26.8 |
| Slightly | 16 | 21.6 | 22 | 26.8 | 21 | 28.4 | 17 | 20.7 |
| Moderately | 16 | 21.6 | 17 | 20.7 | 19 | 25.7 | 24 | 29.3 |
| Severely | 4 | 5.4 | 4 | 4.9 | 2 | 2.7 | 5 | 6.1 |
| Extremely | 0 | 0 | 0 | 0 | 0 | 0 | 0 | 0 |
| Missing | 7 | 9.5 | 7 | 8.5 | 6 | 8.1 | 14 | 17.1 |
|  |  |  |  |  |  |  |  |  |

RCC: Regular continence care, which is the waiting-list group.

SCC: Smart Continence care, which is the intervention group.
