# Supplementary material for: Smart Continence Care for People With Profound Intellectual and Multiple Disabilities Within Dutch Residential Care Facilities: Economic Evaluation Alongside a Cluster Randomized Trial
Source: J Med Internet Res. 2025 Oct 10;27:e72017. doi: 10.2196/72017 (PMC12552815; doi:10.2196/72017)
Supplement: Multimedia Appendix 7 [file jmir_v27i1e72017_app7.docx]

**Disclaimer:** The table below provides an overview of resource use and corresponding costs per variable without the correction for baseline differences. When consulting these numbers, it is important to realize this, as there is an important baseline difference in time spent on continence care. Although the table would suggest that there is a difference in time spent by caregivers on continence care, with the RCC group spending on average 53 hours 12 per week per participant and the SCC group spending on average 40 hours 12 per week per participant, we cannot conclude that SCC is a ‘timesaving’ intervention. Previous analyses found no significant effect for this variable [1]. This can also be observed when comparing the reduction in time spent between the baseline measure (T0) and after 12 weeks (T2). Both groups showed a reduction in weekly time spent on continence care (RCC group -61 minutes, SCC group -53 minutes) at T2 compared to T0.

Table S1: Base case estimate resource use and costs over 12 weeks, based on the bootstrapped (2000x) and imputed (10x) data analysis, no baseline correction. All data is reported in EUROS. Conversion rate of EUR €1=US $1.05469 (as of December 6, 2024)

| **Category** | | | | | | **Resource use** | | **Costs, €** | | **Mean difference costs, €** |
| --- | --- | --- | --- | --- | --- | --- | --- | --- | --- | --- |
|  | | | | **unit** | **Unit price, €** | **RCC group** | **SCC group** | **RCC group** | **SCC group** |  |
|  | | | | | | **Mean (SE)** | | **Mean (SE)** | | **Estimate (95% CI)** |
| **Societal Costs** | | | | | | | | | | |
|  | **Total Healthcare costs** | | | | | | | | | |
|  |  | **Intervention costs for continence care** | | | | | | | | |
|  |  |  | Staff providing CC | Hour | 36.74 | 53.06 (2.85) | 39.67 (2.62) | 1,950 (104.78) | 1,457 (96.42) | -492 (-762.61 to -209.90) |
|  |  |  | Incontinence material | IMC | 0.74 - 1.30^a^ | 220.17 (7.08) | 192.01 (6.54) | 163 (6.80) | 250 (6.38) | 87 (68.60 to 104.57) |
|  |  |  | Disposables | IMC | 0.37 | 220.17 (7.08) | 192.01 (6.54) | 81 (2.62) | 71 (2.42) | -10 (-17.70 to -3.52) |
|  |  |  | Wound and skincare, during continence care | Event | 0.27 | 5.48 (1.52) | 5.94 (1.35) | 4.00 (1.11) | 4.33 (0.99) | 0.33 (-2.35 to 3.20) |
|  |  |  | Laundry bed full | Event | 2.58 | 8.38 (1.53) | 14.72 (1.41) | 22 (3.97) | 38 (3.63) | 16 (6.69 to 26.66) |
|  |  |  | Laundry half bed | Event | 1.29 | 11.36 (1.68) | 13.58 (1.54) | 15 (2.16) | 18 (1.98) | 3 (-2.94 to 8.46) |

| **Category** | | | | | | **Resource use** | | **Costs, €** | | **Mean difference costs, €** |
| --- | --- | --- | --- | --- | --- | --- | --- | --- | --- | --- |
|  | | | | **unit** | **Unit price, €** | **RCC group** | **SCC group** | **RCC group** | **SCC group** |  |
|  | | | | | | **Mean (SE)** | | **Mean (SE)** | | **Estimate (95% CI)** |
|  |  |  | Laundry cloths | Event | 0.86 | 14.67 (2.05) | 19.09 (1.87) | 13 (1.764) | 16 (1.607) | 4 (-0.83 to 8.55) |
|  |  |  | License fee SCC | Day | 6.05 | NA | 84.000 (0.00) | NA | 508.200 (0.000) |  |
|  |  |  | Relays | Day | 0.56 | NA | 84.000 (0.00) | NA | 47.040 (0.000) |  |
|  |  |  | Clip | Day | 0.22 | NA | 84.000 (0.00) | NA | 18.480 (0.000) |  |
|  |  | **Sub-total intervention costs continence care (D):** | | | | | | **2,247 (111.02)** | **2,428 (102.22)** | **181 (-103.99 to 482.18)** |
|  |  | **Other healthcare costs^b^** | | | | | |  |  |  |
|  |  |  | Stay disability care | days | 319.78^c^ | 84.00 (0.00) | 84.00 (0.00) | 26,862 (0.00) | 26,862 (0.00) |  |
|  |  |  | Physiotherapist | visit | 38.89 | 3.06 (0.77) | 2.34 (0.78) | 119 (30.05) | 91 (30.13) | -28 (-115.74 to 66.72) |
|  |  |  | Behavioral therapist | visit | 121 | 0.64 (0.21) | 0.56 (0.20) | 78 (25.86) | 68 (23.97) | -10 (-77.27 to 56.19) |
|  |  |  | General practitioner | visit | 43.31 | 1.42 (0.25) | 0.80 (0.24) | 61 (11.01) | 35 (10.46) | -27 (-54.41 to 0.63) |
|  |  |  | Medical service in-house | visit | 43.31 | 1.73 (0.29) | 0.80 (0.25) | 75 (12.50) | 35 (11.02) | -40 (-72.31 to -8.55) |
|  |  |  | Occupational | visit | 24.32 | 0.29 (0.15) | 0.40 (0.15) | 6.93 (3.733) | 9.62 (3.594) | 2.68 (-5.82 to 14.17) |
|  |  |  | Speech therapist | visit | 40.93 | 0.26 (0.08) | 0.11 (0.07) | 11 (3.11) | 4.63 (3.02) | -6.01 (-14.84 to 2.10) |
|  |  |  | Other therapists | visit | Various prices^d^ | 0.45 (0.42) | 1.20 (0.42) | 18 (19.48) | 54 (19.06) | 36 (-5.91 to 101.54) |
|  |  |  | Outpatient clinic | visit | 120 | 0.31 (0.10) | 0.17 (0.09) | 37 (11.62) | 21 (11.05) | -16 (-49.05 to 16.20) |
|  |  |  | Hospital day treatment | visit | 335 | 0.09 (0.05) | 0.10 (0.050) | 22 (9.80) | 8 (9.32) | -14 (-43.08 to 13.40) |
|  |  |  | Days in hospital | days | 644 | 0.35 (0.14) | 0.04 (1.12) | 225 (91.146) | 29 (79.09) | -197 (-431.45 to -18.13) |
|  |  |  | Ambulance | ride | 528 | 0.04 (0.02) | 0.02 (0.02) | 24 (10.87) | 9 (10.82) | -14 (-44.15 to 11.47) |
|  |  |  | Emergency room | visit | 258 | 0.044 (0.02) | 0.019 (0.02) | 11 (5.40) | 5 (5.55) | -6 (-21.20 to 5.79) |
|  |  | **Sub-total other healthcare costs (E):** | | | | | | **27,556 (127.29)** | **27,226 (114.87)** | **-330 (-675.67 to -35.38)** |
|  | **Total healthcare costs (B):** | | | | | | | **29,767 (168.32)** | **29,664 (152.60)** | **-103 (-557.47 to 390.38)** |

| **Category** | | | | | **Resource use** | | **Costs, €** | | **Mean difference costs, €** |
| --- | --- | --- | --- | --- | --- | --- | --- | --- | --- |
|  | | | **unit** | **Unit price, €** | **RCC group** | **SCC group** | **RCC group** | **SCC group** |  |
|  | | | | | **Mean (SE)** | | **Mean (SE)** | | **Estimate (95% CI)** |
|  | **Costs for participants and their families** | | | | | |  |  |  |
|  |  | Travel costs therapists in-house^e^ | visit | - | ^f^ | ^f^ |  |  |  |
|  |  | Travel costs other | visit | Various^g^ | ^f^ | ^f^ | 5 (15.18) | 35 (14.65) | 30 (-2.34 to 79.70) |
|  |  | Travel costs to outpatient clinics | visit | 30.43 | ^f^ | ^f^ | 9.34 (2.95) | 5.25 (2.80) | -4.09 (-12.44 to 4.11) |
|  |  | Travel costs hospital day treatment | visit | 30.43 | ^f^ | ^f^ | 2.03 (0.89) | 0.77 (0.85) | -1.26 (-3.91 to 1.22) |
|  |  | Travel costs hospital stay | visit | 30.43 | 0.044 (0.02) | 0.001 (0.02) | 1.35 (0.52) | 0.036 (0.48) | -1.31 (-2.97 to 0.00) |
|  |  | Travel costs emergency room | visit | 30.43 | ^f^ | ^f^ | 1.35 (0.64) | 0.59 (0.65) | -0.76 (-2.50 to 0.68) |
|  |  | Productivity loss ^h^ | NA | 0.00 | 0.00 | 0.00 | 0.00 | 0.00 |  |
|  | **Sub-total costs for participants and their family (C):** | | | | | | **19 (16.60)** | **42.17 (15.98)** | **23 (-13.94 to 77.34)** |
| **Total societal costs (A):** | | | | | | | **29,775**  **(171.92)** | **29,707 (156.01)** | **-68 (-574.14 to 466.10)** |
| CC: Continence care, IMC: Incontinence material change, NA: Not applicable, RCC: regular continence care, SCC: smart continence care  ^a^ Regular incontinence material has an average price of €0,74. Smart incontinence material has an average price of €1,30.  ^b^ Medication, besides wound and skincare during continence care, is excluded from this study.  ^c^ Costs for continence care are excluded, see supplementary file 1 for calculation.  ^d^ Various prices apply, see supplementary file 1 for specification.  ^e^ This includes physiotherapist, behavioral therapist, general therapist, medical service, occupational therapist, speech therapist, and other therapists.  ^f^ For resource use, the number of visits to the corresponding variable.  ^g^ See the supplementary file 1 for elaboration.  ^h^ Productivity loss is not applicable to persons with profound intellectual and multiple disabilities. | | | | | | | | | |

Reference

van Cooten VJ, Gielissen MF, den Hollander W, van Mastrigt GA, Smeets O, Bongers IM, et al. Effectiveness of Smart Continence Care for People With Profound Intellectual and Multiple Disabilities: Cluster Randomized Trial. J Med Internet Res. 2025;27:e66389.
